# Effect of Nocturnal Oxygen Therapy on Nocturnal Hypoxemia and Sleep Apnea Among Patients With Chronic Obstructive Pulmonary Disease Traveling to 2048 Meters: A Randomized Clinical Trial

**DOI:** 10.1001/jamanetworkopen.2020.7940

**Published:** 2020-06-22

**Authors:** Lu Tan, Tsogyal D. Latshang, Sayaka S. Aeschbacher, Fabienne Huber, Deborah Flueck, Mona Lichtblau, Stefanie Ulrich, Elisabeth D. Hasler, Philipp M. Scheiwiller, Silvia Ulrich, Konrad E. Bloch, Michael Furian

**Affiliations:** 1 Sleep Disorders Center, Department of Respiratory Medicine, University Hospital of Zurich, Zurich, Switzerland

## Abstract

**Importance:**

There are no established measures to prevent nocturnal breathing disturbances and other altitude-related adverse health effects (ARAHEs) among lowlanders with chronic obstructive pulmonary disease (COPD) traveling to high altitude.

**Objective:**

To evaluate whether nocturnal oxygen therapy (NOT) prevents nocturnal hypoxemia and breathing disturbances during the first night of a stay at 2048 m and reduces the incidence of ARAHEs.

**Design, Setting, and Participants:**

This randomized, placebo-controlled crossover trial was performed from January to October 2014 with 32 patients with COPD living below 800 m with forced expiratory volume in the first second of expiration (FEV_1_) between 30% and 80% predicted, pulse oximetry of at least 92%, not requiring oxygen therapy, and without history of sleep apnea. Evaluations were performed at the University Hospital Zurich (490 m, baseline) and during 2 stays of 2 days and nights each in a Swiss Alpine hotel at 2048 m while NOT or placebo treatment was administered in a randomized order. Between altitude sojourns, patients spent at least 2 weeks below 800 m. Data analysis was performed from January 1, 2015, to December 31, 2018.

**Intervention:**

During nights at 2048 m, NOT or placebo (room air) was administered at 3 L/min by nasal cannula.

**Main Outcomes and Measures:**

Coprimary outcomes were differences between NOT and placebo intervention in altitude-induced change in mean nocturnal oxygen saturation (SpO_2_) as measured by pulse oximetry and apnea-hypopnea index (AHI) measured by polysomnography during night 1 at 2048 m and analyzed according to the intention-to-treat principle. Further outcomes were the incidence of predefined ARAHE, other variables from polysomnography results and respiratory sleep studies in the 2 nights at 2048 m, clinical findings, and symptoms.

**Results:**

Of the 32 patients included, 17 (53%) were women, with a mean (SD) age of 65.6 (5.6) years and a mean (SD) FEV_1_ of 53.1% (13.2%) predicted. At 490 m, mean (SD) SpO_2_ was 92% (2%) and mean (SD) AHI was 21.6/h (22.2/h). At 2048 m with placebo, mean (SD) SpO_2_ was 86% (3%) and mean (SD) AHI was 34.9/h (20.7/h) (*P* < .001 for both comparisons). Compared with placebo, NOT increased SpO_2_ by a mean of 9 percentage points (95% CI, 8-11 percentage points; *P* < .001), decreased AHI by 19.7/h (95% CI, 11.4/h-27.9/h; *P* < .001), and improved subjective sleep quality measured on a visual analog scale by 9 percentage points (95% CI, 0-17 percentage points; *P* = .04). During visits to 2048 m or within 24 hours after descent, 8 patients (26%) using placebo and 1 (4%) using NOT experienced ARAHEs (*P* < .001).

**Conclusions and Relevance:**

Lowlanders with COPD experienced hypoxemia, sleep apnea, and impaired well-being when staying at 2048 m. Because NOT significantly mitigated these undesirable effects, patients with moderate to severe COPD may benefit from preventive NOT during high altitude travel.

**Trial Registration:**

ClinicalTrials.gov Identifier: NCT02150590

## Key Points

**Question:**

Can nocturnal oxygen therapy prevent hypoxemia and sleep apnea among lowlanders with chronic obstructive pulmonary disease when traveling to high altitude?

**Findings:**

In this randomized crossover trial of 32 lowlanders with chronic obstructive pulmonary disease, nocturnal oxygen therapy improved their mean nocturnal oxygen saturation and apnea-hypopnea index during a night at 2048 m. Nocturnal oxygen therapy also reduced the incidence of altitude-induced adverse health effects requiring medical treatment or descent to lower altitude by 85% compared with placebo.

**Meaning:**

Patients with chronic obstructive pulmonary disease may benefit from nocturnal oxygen therapy during travel to high altitude because it reduces nocturnal hypoxemia, sleep disordered breathing, and other adverse health effects.

## Teaser

This randomized clinical trial evaluates whether nocturnal oxygen therapy (NOT) prevents nocturnal hypoxemia and breathing disturbances among lowlanders with chronic obstructive pulmonary disease (COPD) in the first night of a stay at 2048 m and reduces the incidence of altitude-related adverse health effects.

## Introduction

Chronic obstructive pulmonary disease (COPD) is characterized by chronic airflow limitation related to airway inflammation associated with airway narrowing and destruction of the lung parenchyma. Given that COPD is highly prevalent and the fourth leading cause of death, it is a major health problem worldwide.^1^ Although COPD is a progressive condition, medical treatment may improve symptoms in many patients. Today, millions of tourists travel to high altitude for leisure or work activities, and many patients with COPD are among them. Studies have found that acute ascent to altitude above 2500 m causes hypoxemia, periodic breathing, disturbed sleep, and exercise intolerance in healthy travelers,^2,3^ whereas short-term altitude exposures between 2000 m and 2500 m (equivalent to pressurized airplane cabins during long-distance flights) seem to be unproblematic for healthy individuals. However, patients with preexisting lung diseases, such as COPD, may experience severe hypoxemia at high altitude because of disease-related ventilatory limitations and impaired gas exchange.^4,5,6,7,8^ Thus, in a randomized clinical trial among patients with moderate to severe COPD (ie, forced expiratory volume in the first second of expiration [FEV_1_] between 30% and 80% predicted) who were exposed for 2 days at 2590 m,^4^ we observed an incidence of 24% of altitude-related adverse health effects (ARAHEs) requiring medical intervention. Moreover, endurance of submaximal exercise at this altitude was reduced by more than half compared with near sea level,^5^ and the patients with COPD experienced nocturnal hypoxemia, frequent sleep apnea,^7^ and autonomic dysregulation.^8^ In a randomized, placebo-controlled trial among 118 lowlanders with COPD,^6^ preventive dexamethasone treatment improved nocturnal hypoxemia and sleep apnea during a stay at 3100 m; however, dexamethasone did not reduce the incidence of ARAHEs and induced hyperglycemia. Supplemental oxygen therapy may improve oxygenation and exercise capacity in patients with COPD who are acutely exposed to high altitude.^9,10^ While daytime oxygen administration during mountain outdoor activities is inconvenient or may be unpractical, nocturnal oxygen therapy (NOT) could be a feasible way to prevent severe hypoxemia, breathing disturbances, and ARAHEs in patients with COPD during altitude sojourns. Therefore, the purpose of this randomized, placebo-controlled trial was to evaluate the hypothesis that NOT would improve nocturnal oxygen saturation and breathing instability as well as reduce the incidence of ARAHEs in lowlanders with COPD staying at high altitude.

## Methods

### Study Design

This randomized, placebo-controlled, crossover trial was performed from January 1 to October 31, 2014, in patients with COPD living below 800 m. During a stay at 2048 m, it evaluated the effects of NOT on the primary outcomes, ie, nocturnal arterial oxygen saturation (SpO_2_) as measured by pulse oximetry and breathing disturbances, as well as on several secondary outcomes, including sleep and ARAHEs. Participants underwent baseline evaluations during 1 day and night at 490 m (Zurich, Switzerland) and had the same evaluations during the course of 2 visits, of 2 days and nights each, in a mountain hotel at 2048 m (St Moritz, Switzerland). In the nights at 2048 m patients received either NOT or placebo (ie, ambient air) according to a randomized crossover trial design. Patients had to spend at least 2 weeks at low altitude (ie, <800 m) between study periods. The protocol was approved by the cantonal ethics committee Zurich and is available in Supplement 1. Participants gave written informed content; there was no financial compensation. The trial followed the Consolidated Standards of Reporting Trials (CONSORT) reporting guideline.

### Patients

Patients with COPD with Global Initiative for Obstructive Lung Disease grades 2 to 3 (ie, FEV_1_ / forced vital capacity, <0.7; FEV_1_, 30% to 80% predicted), aged 18 to 75 years, both sexes, and living at low altitude (ie, <800 m) were invited. Exclusion criteria were hypoxemia of SpO_2 _lower than 92% at 490 m, home oxygen or CPAP therapy, any uncontrolled cardiovascular disease, or history of obstructive sleep syndrome. The 5 patients in whom clinical evaluation raised the suspicion of undiagnosed sleep apnea were evaluated by nocturnal pulse oximetry and were excluded if their oxygen desaturation index exceeded 15 events/h. No patients were excluded. Further exclusion criteria are described in the eAppendix in Supplement 2.

### Randomization and Intervention

**Figure 1.**
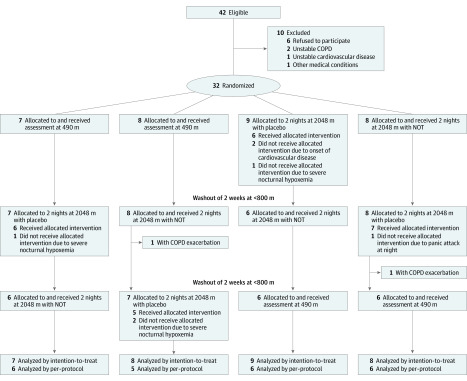
Study Flow Diagram Altitude allocation sequence and order of intervention (oxygen or placebo) at 2048 m were randomized. After evaluations at 2048 m, a 2-week washout period below 800 m was applied to avoid carry-over effects. COPD indicates chronic obstructive pulmonary disease.

Patients were randomized in balanced blocks of 4 and were assigned to sequences of altitude exposure and treatment (1-4) by letting them draw a sealed envelope with 1 of the following allocations: (1) assessment at 490 m first, allocated to receive placebo on first trip to 2048 m, then NOT on second trip; (2) assessment at 490 m first, allocated to receive NOT on first trip to 2048 m, then placebo on second trip; (3) allocated to receive placebo on first trip to 2048 m, then NOT on second trip, with assessment at 490 m last; and (4) allocated to receive NOT on first trip to 2048 m, then placebo on second trip, with assessment at 490 m last (Figure 1). Participants traveled by train and automobile within 3 hours from 490 to 2048 m. Between altitude trips, a washout period of at least 2 weeks at lower than 800 m was interposed. During 2 nights at 2048 m, patients wore nasal prongs connected to a concentrator (EverFlow, Philips Respironics) delivering oxygen or placebo (ambient air) at a flow rate of 3 L/min. Patients were masked for the intervention by placing concentrators in a separate room. Investigators performing the data analysis were also masked to intervention and exposure sequence. For safety reasons, patients’ nocturnal oxygen saturation was monitored by investigators, making masking of these individuals not feasible.

### Assessments

In the first night of each visit, polysomnography (Alice 5, Philips Respironics) was performed, including transcutaneous capnography and cerebral tissue oxygen saturation monitoring by near-infrared spectroscopy as described previously (eAppendix in Supplement 2).^11,12,13^ On the second night at 2048 m, respiratory sleep studies without neurophysiologic channels and near-infrared spectroscopy were conducted. Sleep stages and arousals were scored according to the Rechtschaffen and Kales standard^14^ and American Academy of Sleep Medicine guidelines.^12^ Scoring of apneas and hypopneas has been described previously (eAppendix in Supplement 2).^7^ The apnea-hypopnea index (AHI) was computed as the number of events per hour of total sleep time and time in bed, and the oxygen desaturation index (>3% SpO_2_ dips) was computed as the number of events per hour of time in bed.

Morning evaluations included weight, heart rate, blood pressure, and SpO_2_. Sleepiness was evaluated by the Karolinska sleepiness scale^15^ and the Stanford sleepiness scale.^16^ Subjective sleep quality was rated on a 100-mm visual analog scale ranging from 0 (very bad) to 100 (excellent).^6^ Symptoms of acute mountain sickness (AMS) were evaluated by the Environmental Symptoms Questionnaire Cerebral score. Scores of at least 0.7 on the Environmental Symptoms Questionnaire Cerebral score were considered to reflect clinically relevant AMS.^17^ During the psychomotor vigilance test, patients were sitting comfortably in a quiet room. They had to press a button on a handheld device in response to a light on the device flashing at random intervals, and median reaction time was recorded.^18^ Arterial blood gas analysis, 6-minute walk testing, and lung function testing, including single-breath diffusing capacity for carbon monoxide, were performed in the morning during ambient air breathing (eAppendix in Supplement 2).^19,20,21^

### Outcomes

Coprimary outcomes were differences between NOT vs placebo treatment in mean nocturnal SpO_2_ and AHI from 490 m to the first night at 2048 m. Secondary outcomes were additional variables derived from sleep studies in the 2 nights at 2048 m, arterial blood gases, lung function, cognitive performance, and the incidence of ARAHEs. According to predefined safety precautions, patients experiencing any ARAHE, including intercurrent illness (eg, exacerbation of COPD, cardiovascular disease, infection, or new diseases), severe hypoxemia (ie, SpO_2_ <75% for >30 minutes at any time while at high altitude), AMS (Environmental Symptoms Questionnaire Cerebral score, ≥0.7), or any other condition requiring therapy, were treated as appropriate and withdrawn from the study.

### Sample Size

We aimed to detect differences between NOT and placebo in mean (SD) SpO_2_ of 2% (4%) and in the mean (SD) AHI of 10/h (20/h) with a 2-sided α < .05 and a power of 80%. The required sample size was 32 participants.^7^

### Statistical Analysis

Data analysis was performed from January 1, 2015, to December 31, 2018. The data are summarized as mean and SD. The coprimary outcomes were analyzed by intention-to-treat, with missing data replaced by multiple imputations, and per-protocol.^22^ Data from 4 patients who received NOT because of severe hypoxemia (SpO_2_ <75% for >30 minutes) during the altitude sojourn with placebo were included in the intention-to-treat analysis but eliminated from the per-protocol analysis. Mean values, SEs, mean differences, and 95% CIs between measures at 490 m and at 2048 m with NOT or placebo from continuous coprimary outcomes SpO_2_ and AHI and continuous secondary outcomes were computed using linear mixed models with outcomes as dependent variables and altitude and intervention as independent variables.^23^ The proportion of participants experiencing ARAHEs was evaluated by the Fisher exact test and logistic regression analysis. Exploratory regression analyses were performed to elucidate low-altitude variables associated with ARAHE, breathing instability, and nocturnal SpO_2_ at 2048 m. Statistical significance was assumed when 95% CIs of mean differences did not overlap zero. For secondary outcomes and other exploratory analyses, no adjustment of the significance threshold was performed. Statistical analysis was performed by Stata version 15.1 (StataCorp), and the statistical analysis plan is available in Supplement 1.

## Results

A total of 42 patients were assessed for eligibility, and 32 (17 [53%] women and 15 [47%] men), with a mean (SD) age of 65.6 (5.6) years and a mean (SD) FEV_1_ of 53.1% (13.2%) predicted, were included in the study (Figure 1). The per-protocol analysis included 23 patients. Baseline characteristics are displayed in Table 1. The participants had moderate to severe COPD, more than half (17 [53%]) had cardiovascular comorbidities (mainly arterial hypertension and coronary heart disease), and they were not excessively sleepy, ie, had normal Epworth sleepiness scores (mean [SD], 4.4 [3.3]).

**Table 1.** Patient Characteristics

Variable	Population, No. (%)	
	Intention to treat (N = 32)	Per protocol (n = 23)
Men	15 (47)	10 (43)
Women	17 (53)	13 (57)
Age, mean (SD), y	65.6 (5.6)	66.0 (5.1)
Smoking		
Current	9 (28)	4 (17)
Pack-years, mean (SD)	37.6 (31.5)	38.2 (31.5)
FEV_1_, % predicted, mean (SD)	53.1 (13.2)	53.7 (12.8)
GOLD grade 2	23 (72)	16 (70)
Body mass index, mean (SD)^a^	26.0 (4.5)	25.3 (3.9)
Epworth sleepiness scale score, mean (SD)	4.4 (3.3)	4.6 (3.5)
Comorbidities		
Cardiovascular disease including hypertension	17 (53)	12 (52)
Diabetes	4 (13)	3 (13)
Depression	5 (16)	3 (13)
Medication		
Inhaled glucocorticosteroids	5 (16)	2 (9)
Inhaled β-adrenergics	24 (75)	17 (74)
Inhaled anticholinergics	23 (72)	18 (78)
Diuretics	5 (16)	2 (9)
Antihypertensive medication	17 (53)	12 (52)
Antidiabetics	4 (13)	3 (13)
Antidepressants	5 (16)	3 (13)

Abbreviations: FEV_1_, forced expiratory volume in the first second of expiration; GOLD, Global Initiative for Obstructive Lung Disease.

^a^ Body mass index calculated as weight in kilograms divided by height in meters squared.

### Arterial Oxygenation and AHI

**Figure 2.**
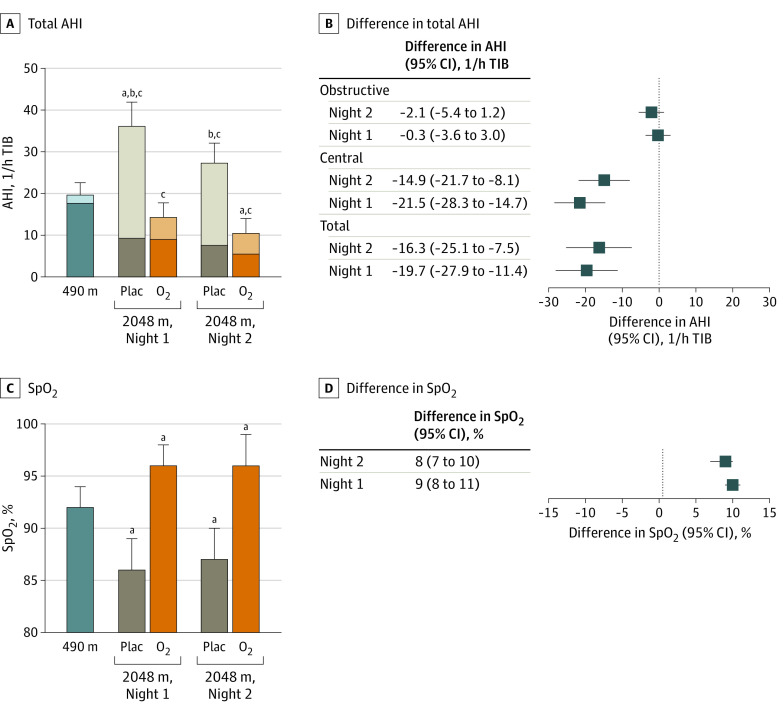
Effect of Altitude and Nocturnal Oxygen Therapy on the Apnea-Hypopnea Index (AHI) and Mean Arterial Oxygen Saturation (SpO_2_) in Intention-to-Treat Analysis Central proportions of AHI are indicted by the lighter shades; darker shades, the obstructive proportion of the apnea-hypopnea index; whiskers, 95% CI; O_2_, oxygen; plac, placebo; and TIB, time in bed. ^a^*P* < .05 compared with 490 m in total AHI or mean SpO_2_. ^b^*P* < .05 compared with 490 m in central AHI. ^c^*P* < .05 compared with 490 m in obstructive AHI.

The results of the intention-to-treat analysis of the primary outcomes are illustrated in Figure 2, and the results of the per-protocol analysis are summarized in Table 2. During both nights at 2048 m with placebo, the mean (SD) nocturnal SpO_2_ was significantly decreased (night 1: 86% [3%]; night 2: 87% [3%]) compared with values at 490 m (night 1: 92% [2%]; *P* < .001) (Table 2 and Figure 2). The absolute mean treatment effects during night 1 and 2 with NOT at 2048 m were 9 (95% CI, 8 to 11) percentage points and 8 (95% CI, 7 to 10) percentage points, respectively, in the intention-to-treat analysis (Figure 2). During both nights at 2048 m with placebo, the mean (SD) total AHI was significantly increased (night 1: 34.9/h [20.7/h]; night 2: 27.8/h [21.0/h]) compared with values at 490 m (nights 1: 21.6/h [22.2/h]; *P* < .001) because of a major increase in the central AHI (night 1: difference, 24.9/h; 95% CI, 18.7/h to 31.1/h, *P* < .001) (Table 2 and Figure 2), while obstructive events were slightly decreased in comparison with 490 m (night 1 difference, −0.3/h; 95% CI, −3.6/h to 3.0/h). With NOT during night 1 and 2 at 2048 m, the mean treatment effects in total AHI were −19.7/h (95% CI, −27.9/h to −11.4/h) and −16.3/h (95% CI, −25.1/h to −7.5/h), respectively, in the intention-to-treat analysis (Figure 2).

**Table 2.** Results of Sleep Studies in Per-Protocol Analysis^a^

Outcome	Mean (SD)					NOT vs placebo at 2048 m, mean difference (95% CI)	
	First night at 490 m	2048 m					
		With nocturnal placebo		With NOT			
		First night	Second night	First night	Second night	First night	Second night
Time in bed, min	497 (29)	486 (25)	476 (60)	484 (49)	492 (25)	−2 (−23 to 20)	16 (−5 to 37)
Nocturnal SpO_2_, %	92 (2)	86 (3)^b^	87 (3)^b^	96 (2)^b^	96 (3)^b^	10 (9 to 11)^c^	9 (7 to 10)^c^
SpO_2 _<90%, % TIB	12.5 (19.0)	77.4 (27.7)^b^	70.0 (30.5)^b^	1.2 (2.8)^b^	4.5 (20.6)	−76.4 (−87.5 to −65.3)^c^	−65.6 (−76.7 to −54.4)^c^
ODI, 1/h TIB^d^	9.3 (11.7)	40.0 (28.4)^b^	34.1 (24.4)^b^	4.1 (10.3)	4.2 (11.2)	−35.9 (−44.4 to −27.5)^c^	−29.9 (−38.3 to −21.4)^c^
Total AHI, 1/h TIB	19.7 (13.9)	36.1 (27.9)^b^	27.3 (22.6)^b^	14.3 (16.9)	10.4 (10.4)^b^	−21.7 (−29.3 to −14.3)^c^	−17.0 (−24.4 to −9.5)^c^
Obstructive AHI, 1/h TIB	17.7 (13.1)	9.3 (11.2)^b^	7.6 (8.8)^b^	9.0 (10.9)^b^	5.5 (6.9)^b^	−0.3 (−3.6 to 3.0)	−2.1 (−5.4 to 1.2)
Central AHI, 1/h TIB	2.1 (2.0)	26.8 (23.9)^b^	19.7 (19.5)^b^	5.3 (11.4)	4.8 (7.4)	−21.5 (−28.3 to −14.7)^c^	−14.9 (−21.7 to −8.1)^c^
Periodic breathing, % TIB	0.2 (0.6)	15.5 (16.6)^b^	12.1 (13.1)^b^	3.2 (8.5)	3.1 (5.5)	−12.4 (−17.1 to −7.7)^c^	−9.0 (−13.7 to −4.2)^c^
TcCO_2_, kPa	5.5 (0.7)	4.8 (0.4)^b^	4.8 (0.4)^b^	4.9 (0.8)^b^	5.3 (0.7)	0.3 (−0.1 to 0.5)	0.4 (0.1 to 0.8)^c^
Heart rate, bpm	69 (10)	76 (12)^b^	74 (12)^b^	68 (12)	70 (10)	−7 (−10 to −5)^c^	−4 (−6 to −1)^c^
VES, 1/h TIB	10.2 (28.3)	14.5 (27.7)	18.8 (44.5)	9.7 (25.8)	6.4 (13.7)	−4.8 (−14.9 to 8.4)	−12.3 (−22.6 to −2.1)^c^
SVES, 1/h TIB	10.3 (18.2)	25.4 (75.3)	7.7 (14.7)	15.6 (39.5)	19.6 (44.9)	−9.8 (−27.2 to 7.6)	12.3 (−5.3 to 29.9)
Total sleep time, min	400 (61)	349 (76)^b^	NA	386 (71)	NA	35 (9 to 61)^c^	NA
Total AHI, 1/h TST	22.0 (15.8)	44.0 (35.8)^b^	NA	16.9 (19.7)	NA	−27.1 (−36.6 to −17.6)^c^	NA
Obstructive AHI, 1/h TST	20.4 (15.2)	11.6 (13.2)^b^	NA	11.2 (12.5)^b^	NA	−0.5 (−4.7 to 3.7)	NA
Central AHI, 1/h TST	1.6 (1.9)	32.4 (32.3)^b^	NA	5.7 (13.7)	NA	−26.7 (−35.2 to −18.1)^c^	NA
Nocturnal CTO, %^e^	64 (6)	59 (5)^b^	NA	69 (6)^b^	NA	9 (7 to 11)^c^	NA
cODI, 1/h TIB^f^	0.8 (1.3)	4.2 (5.6)^b^	NA	0.6 (1.1)	NA	−3.6 (−5.8 to −1.5)^c^	NA
Sleep efficiency, % TIB	81 (11)	72 (15)^b^	NA	78 (14)	NA	7 (2 to 11)^c^	NA
WASO, min	75.3 (49.3)	116.8 (67.8)^b^	NA	86.3 (62.0)	NA	−30.0 (−52.2 to −7.9)^c^	NA
Sleep latency, min	21.2 (24.1)	19.6 (16.0)	NA	20.6 (15.2)	NA	0.8 (−6.7 to 8.2)	NA
Arousal index, /h	13.7 (8.8)	18.7 (16.2)^b^	NA	13.8 (9.7)	NA	−4.6 (−9.2 to 0)^c^	NA
NREM 1, % TST	29.9 (15.0)	33.0 (21.2)	NA	25.5 (18.4)	NA	−7.4 (−14.0 to −0.8)^c^	NA
NREM 2, % TST	40.6 (12.4)	42.0 (16.8)	NA	45.6 (13.2)	NA	3.6 (−1.6 to 8.9)	NA
NREM 3 and 4, % TST	14.1 (6.7)	13.2 (10.1)	NA	13.8 (8.9)	NA	0.1 (−3.3 to 3.5)	NA
REM, % TST	15.4 (4.3)	11.9 (6.8)^b^	NA	15.1 (6.8)	NA	3.2 (0.7 to 5.7)^c^	NA

Abbreviations: AHI, apnea-hypopnea index; bpm, beats per minute; CTO, cerebral tissue oxygenation; cODI, cerebral ODI; NA, not applicable; NOT, nocturnal oxygen therapy; NREM, non–rapid eye movement sleep; ODI, oxygen desaturation index; REM, rapid eye movement sleep; SpO_2_, mean nocturnal arterial oxygen saturation by pulse oximetry; SVES, supraventricular extra-beats; TcCO_2_, transcutaneous capnography; TIB, time in bed; TST, total sleep time; VES, ventricular extra-beats; WASO, wake after sleep onset.

^a^ In the first night at all locations, full polysomnography was performed; in the second night, respiratory sleep studies were performed. Thus, sleep variables are not available for second nights.

^b^ *P* < .05 altitude vs 490 m.

^c^ *P* < .05 NOT vs placebo.

^d^ ODI defined as greater than 3% dips in SpO_2_.

^e^ CTO measured by near-infrared spectroscopy.

^f^ cODI defined as greater than 3% dips in CTO.

### Other Indices of Nocturnal Breathing

Correspondingly, in the per-protocol analysis, NOT at 2048 m prevented the altitude-induced increase in central AHI (difference on night 1, −21.5/h; 95% CI, −28.3/h to −14.7/h), periodic breathing (difference on night 1, −12.4% of time in bed; 95% CI, −17.1% to −7.7% of time in bed), oxygen desaturation index (difference on night 1, −35.9/h; 95% CI, −44.4/h to −27.5/h), time spent with SpO_2 _lower than 90% (difference on night 1, −76.4% of time in bed; 95% CI, −87.5% to −65.3% of time in bed), and cerebral tissue deoxygenation (difference on night 1, 9 percentage points; 95% CI, 7 percentage points to 11 percentage points) (Table 2). Analysis of groups allocated to different altitude and drug exposure sequences revealed no order effect for the coprimary outcomes (eTable 1 and eTable 2 in Supplement 2).

There was an altitude-induced decrease in transcutaneous carbon dioxide in nights with placebo at 2048 m compared with 490 m (first night: mean [SD], 4.8 [0.4] kPa vs 5.5 [0.7] kPa; *P* < .001); with NOT, a similar decrease was seen on the first but not on the second night at 2048 m compared with 490 m (mean [SD], 4.9 [0.8] kPa vs 5.5 [0.7] kPa; *P* = .01). With placebo, a significant increase in heart rate during nights at 2048 m was observed compared with 490 m (first night: mean [SD] 76 [12] beats/min [bpm] vs 69 [10] bpm; *P* < .001), but NOT reduced heart rate by −7 bpm (95% CI, −10 to −5 bpm) in night 1 and −4 bpm (95% CI, −6 to −1 bpm) in night 2 compared with placebo.

### Sleep Indices

Compared with values at 490 m, receiving a placebo at 2048 m was associated with a decrease in total sleep time (mean [SD], 400 [61] min vs 349 [76] min; *P* < .001), proportion of rapid-eye movement sleep (mean [SD], 15.4% [4.3%] vs 11.9% [6.8%]; *P* = .004), and sleep efficiency (mean [SD], 81% [11%] of time in bed vs 72% [15%] of time in bed; *P* < .001), and an increase in wake time after sleep onset (mean [SD], 75.3 [49.3] min vs 116.8 [67.8] min; *P* < .001) and arousal index (mean [SD], 13.7/h [8.8/h] vs 18.7/h [16.2/h]; *P* = .03). NOT improved total sleep time (difference on night 1, 35 min; 95% CI, 9 to 61 min), proportion of rapid-eye movement sleep (difference on night 1, 3.2% of total sleep time; 95% CI, 0.7% to 5.7% of total sleep time), sleep efficiency (difference on night 1, 7% of time in bed; 95% CI, 2% to 11% of time in bed), and reduced wake time after sleep onset (difference on night 1, −30.0 min; 95% CI, −52.2 to −7.9 min) and arousal index (difference on night 1, −4.6/h; 95% CI, −9.2/h to 0.0/h) (Table 2).

### Variables Associated With Nocturnal Pulse Oximetry and AHI

Linear mixed regression models for AHI and nocturnal SpO_2_ at 2048 m under placebo as dependent variables revealed that low altitude AHI and nocturnal SpO_2_ as well as acclimatization (night 2 vs night 1 at 2048 m) were independently associated (eTable 3 and eTable 4 in Supplement 2). Univariable logistic regression analyses evaluating low-altitude variables associated with ARAHE included age, sex, body mass index, lung function outcomes, arterial blood gases, and 6-minute walk distance. However, none of the variables was associated with ARAHEs (eTable 5 in Supplement 2).

### ARAHEs

During altitude sojourns or within the first 24 hours after descent, 8 of 31 patients (26%) using placebo and 1 of 28 patients (4%) using NOT experienced ARAHEs (odds ratio of NOT vs placebo, 0.10; 95% CI 0.01 to 0.88; *P* = .04). Among the 8 patients who received placebo and experienced ARAHEs, 4 (50%) had severe nocturnal hypoxemia (ie, SpO_2_ <75% for >30 minutes), 1 (13%) had a panic attack, 1 (13%) had nocturnal urinary incontinence, 1 (13%) had intolerable dyspnea sensation, 1 (13%) had nonsustained ventricular tachycardia during exercise, and 1 (13%) had a COPD exacerbation. In 3 patients (38%), these ARAHEs were accompanied or followed by clinically relevant AMS. The 1 patient (13%) experiencing an ARAHE while using NOT had a COPD exacerbation on the day of descent from 2048 m.

### Daytime Evaluations

Daytime outcomes are presented in Table 3. After the first night at 2048 m, patients with placebo had higher AMS scores compared with 490 m (mean [SD], 0.2 [0.5] vs 0.1 [0.1]; *P* = .01), increased daytime sleepiness scores (mean [SD], 3.6 [1.9] vs 2.8 [1.4]; *P* = .03), and rated their subjective sleep quality worse (mean [SD], 51% [23%] vs 64% [20%]; *P* = .002) (Table 3). With NOT, patients rated their sleep quality better compared with placebo (difference after the first night, 9 percentage points; 95% CI, 0 to 17 percentage points) (Table 3).

**Table 3.** Daytime Measures for Per-Protocol Analysis

Outcome	Mean (SD)					NOT vs placebo at 2048 m, mean difference (95% CI)	
	490 m Second day	2048 m					
		With nocturnal placebo		With NOT			
		Second day	Third day	Second day	Third day	Second day	Third day
**Questionnaire evaluation**							
AMSc	0.1 (0.1)	0.2 (0.5)^a^	0.1 (0.6)	0.1 (0.3)	0.1 (0.3)	−0.1 (−0.2 to 0.1)	0 (−0.2 to 0.1)
Karolinska sleepiness score	2.8 (1.4)	3.6 (1.9)^a^	2.8 (1.4)	3.4 (1.8)	3.1 (1.8)	−0.2 (−0.9 to 0.5)	0.4 (−0.3 to 1.0)
Subjective sleep quality visual analog scale, %	64 (20)	51 (23)^a^	60 (20)	59 (20)	64 (23)	9 (0 to 17)^b^	3 (−5 to 12)
Estimated time spent awake at night, min	55 (50)	86 (71)^a^	70 (73)	68 (72)	56 (56)	−18 (−48 to 12)	−14 (−45 to 16)
**Clinical examination and exercise performance**							
Weight, kg	70.4 (12.1)	70.4 (12.0)	70.5 (11.9)	70.4 (12.0)	70.9 (12.1)	0 (−0.6 to 0.6)	0.4 (−0.2 to 1.0)
At rest							
Heart rate, bpm	68 (10)	74 (12)^a^	71 (13)^a^	69 (12)	68 (10)	−4 (−7 to −2)^b^	−3 (−5 to 0)^b^
BP, mm Hg							
Systolic	130 (19)	133 (14)	129 (14)	130 (19)	124 (14)^a^	−4 (−9 to 2)	−5 (−10 to 1)
Diastolic	73 (10)	78 (10)^a^	74 (9)	78 (9)^a^	72 (9)	1 (−3 to 5)	−2 (−6 to 1)
6MWT distance, m	543 (89)	486 (94)^a^	493 (97)^a^	480 (118)^a^	492 (107)^a^	−7 (−24 to 11)	−2 (−19 to 16)
At end of 6MWT							
Borg CR10 dyspnea score	3.2 (1.9)	3.8 (2.5)^a^	3.9 (2.0)^a^	4.0 (2.6)^a^	4.0 (2.5)^a^	0.1 (−0.5 to 0.7)	0.1 (−0.5 to 0.7)
Heart rate, bpm	108 (21)	113 (18)	116 (20)^a^	113 (21)	117 (19)^a^	0 (−7 to 6)	2 (−5 to 8)
BP, mm Hg							
Systolic	163 (33)	170 (32)	168 (31)	160 (30)	160 (28)	−10 (−18 to −2)^b^	−8 (−17 to 0)^b^
Diastolic	82 (13)	88 (17)^a^	89 (18)^a^	83 (11)	84 (11)	−6 (−11 to 0)^b^	−5 (−10 to 1)
**Arterial blood gas analysis**							
pH	7.44 (0.02)	7.47 (0.03)^a^	NA	7.47 (0.02)^a^	NA	0.00 (−0.01 to 0.01)	NA
Paco_2_, kPa	5.3 (0.4)	4.5 (0.5)^a^	NA	4.5 (0.5)^a^	NA	0 (−0.1 to 0.3)	NA
Pao_2_, kPa	9.1 (0.9)	7.9 (0.8)^a^	NA	7.9 (0.9)^a^	NA	−0.1 (−0.4 to 0.1)	NA
Sao_2_, %	94 (2)	90 (3)^a^	NA	90 (4)^a^	NA	0 (−1 to 1)	NA
Hemoglobin, g/dL	14.1 (1.2)	14.5 (1.2)^a^	NA	14.6 (1.3)^a^	NA	0 (−0.2 to 0.3)	NA
**Lung function**							
FEV_1_, L	1.7 (0.5)	1.7 (0.5)	1.8 (0.5)^a^	1.7 (0.5)	1.8 (0.5)^a^	0 (0 to 0.1)	0 (−0.1 to 0.1)
FEV_1_, % predicted	63 (14)	61 (14)	66 (14)^a^	64 (12)	66 (14)^a^	2 (−1 to 4)	−1 (−3 to 1)
FVC, L	3.1 (0.7)	3.2 (0.8)^a^	3.3 (0.7)^a^	3.2 (0.8)^a^	3.2 (0.8)	0 (−0.1 to 0.1)	−0.1 (−0.2 to 0)
FVC, % predicted	90 (12)	92 (16)^a^	95 (13)^a^	93 (15)	92 (13)	0 (−3 to 3)	−3 (−6 to 0)^b^
FEV_1_/FVC, %	54 (12)	52 (11)^a^	54 (9)	54 (10)	56 (10)	1 (0 to 3)	1 (−1 to 3)
TLco adjusted for hemoglobin, mL/mm Hg/min	5.8 (1.9)	6.0 (2.0)^a^	6.0 (2.0)^a^	6.2 (2.0)^a^	6.1 (2.1)^a^	0.1 (−0.1 to 0.3)	0 (−0.2 to 0.3)
TLco adjusted for hemoglobin, % predicted	71 (20)	74 (21)	74 (21)	77 (21)	76 (21)	2 (−1 to 5)	0 (−3 to 3)
TLco adjusted for hemoglobin and altitude, % predicted	69 (20)	66 (19)	67 (19)	68 (19)	68 (20)	2 (−1 to 4)	0 (−3 to 3)
**Vigilance**							
PVT reaction time, ms	268 (47)	262 (42)	258 (41)	261 (52)	271 (45)	−1 (−14 to 12)	13 (0 to 26)^b^

Abbreviation: 6MWT, 6-minute walk test; AMSc, acute mountain sickness score; BP, blood pressure; bpm, beats per minute; CR10, category ratio anchored at the number 10; FEV_1_, forced expiratory volume in the first second of expiration; FVC, forced vital capacity; NA, not applicable; NOT, nocturnal oxygen therapy; Paco_2_, partial pressure of arterial carbon dioxide; Pao_2_, partial pressure of arterial oxygen; PVT, psychomotor vigilance test; Sao_2_, arterial oxygen saturation; TLco, diffusing capacity for carbon monoxide.

SI conversion factor: To convert hemoglobin to grams per liter, multiply by 10.

^a^ *P* < .05 altitude vs 490 m.

^b^ *P* < .05 NOT vs placebo.

Compared with 490 m, higher rated dyspnea score (mean [SD], 3.2 [1.9] vs 3.8 [2.5]; *P* = .02) and a shorter 6-minute walk distance (mean [SD], 543 [89] m vs 486 [94] m; *P* < .001) were observed at 2048 m with placebo. Receiving NOT did not change the 6-minute walk distance or dyspnea score compared with placebo but lowered end-exercise systolic blood pressure (difference after first night, −10 mm Hg; 95% CI, −18 to −2 mm Hg) and diastolic blood pressure (difference after first night, −6 mm Hg; 95% CI, −11 to 0 mm Hg) (Table 3).

Arterial blood gas analysis in the morning after the first night at 2048 m revealed higher pH compared with 490 m (mean [SD], 7.47 [0.03] vs 7.44 [0.02]; *P* < .001) and lower partial pressure of arterial oxygen (Pao_2_; mean [SD], 7.9 [0.8] kPa vs 9.1 [0.9] kPa; *P* < .001) and partial pressure of arterial carbon dioxide (Paco_2_; mean [SD], 4.5 [0.5] kPa vs 5.3 [0.4] kPa; *P* < .001) with placebo. With NOT, no changes in arterial blood gases were observed compared with placebo. Lung function and psychomotor vigilance test reaction time were not altered at 2048 m with placebo or NOT compared with 490 m.

## Discussion

We performed a randomized, placebo-controlled, crossover trial to evaluate the effect of NOT on nocturnal breathing, sleep, and daytime performance in lowlanders with grade 2 to 3 COPD staying for 2 days and nights at an altitude of 2048 m. Exposure to high altitude induced arterial and cerebral hypoxemia, breathing instability, worsening of sleep structure, and subjective sleep quality. Furthermore, 26% of patients using placebo experienced ARAHEs requiring medical treatment or descent. We found that NOT prevented hypoxemia, attenuated sleep apnea, improved sleep structure and subjective sleep quality at high altitude, and reduced the incidence of ARAHEs by 85% compared with placebo.

In a previous trial among 40 patients with moderate to severe COPD (median FEV_1_, 59% predicted) ascending to 2590 m,^7^ nocturnal hypoxemia and sleep apnea were similar compared with the current findings (median nocturnal SpO_2_, 85% vs 86%; AHI, 39.5/h vs 36.1/h time in bed). In the cited trial,^7^ half of the patients had an intermediate 2-day altitude stay at 1650 m before ascending to 2590 m. Therefore, acclimatization might have dampened the altitude effect.^7^ At 2590 m, a 24% incidence of ARAHEs was reported,^4^ confirming the susceptibility of patients with COPD to hypobaric hypoxia. For comparison, at altitudes up to 2590 m, adverse events requiring medical treatment or descent were uncommon in healthy volunteers or in patients with obstructive sleep apnea syndrome.^3,24^ Randomized, controlled studies with shorter exposure (ie, a few hours) to hypobaric hypoxia, such as during airplane travel, showed decrements in Pao_2_ and exercise capacity, but patients otherwise remained asymptomatic.^25,26,27^ However, a cross-sectional study^28^ has shown that 12.1% of all in-flight emergencies are due to respiratory-related symptoms, some resulting in admission to the hospital. In 49.9% of in-flight medical emergencies, supplemental oxygen was administered.^28^ Therefore, the British Thoracic Society recommends in-flight oxygen therapy in patients with very severe COPD (ie, FEV_1_ ≤30% predicted) when Pao_2_ is lower than 6.67 kPa or Sao_2_ is lower than 85% during a hypoxia altitude simulation test (ie, breathing fraction of inspired oxygen, 0.15; equivalent to 2438 m for 20 minutes).^29^ However, the accuracy of the hypoxia altitude simulation test in predicting symptoms and level of hypoxemia in patients with COPD during flights is low,^26^ and the test has never been validated for prediction of adverse effects during altitude travel. Patients in the current trial, who would not have required a hypoxia altitude simulation test before traveling by airplane according to the guidelines (FEV_1_ ≥30% predicted), had daytime Pao_2_ measured 18 hours after arrival at 2048 m between 5.7 to 9.0 kPa and Sao_2_ between 79% to 95%, indicating that 5 of 31 patients (16%) with moderate to severe COPD would have fulfilled the British Thoracic Society criteria for oxygen supplementation at an altitude of 2048 m.

Nocturnal hypoxemia and AHI in the current patients with COPD at 2048 m (mean [SD] SpO_2_, 86% [3%]; AHI, 36.1/h [27.9/h] with placebo) were worse compared with corresponding values in 51 healthy volunteers at 2590 m (median [interquartile range {IQR}] SpO_2_, 90% [89%-91%]; median [IQR] AHI, 13.1/h [6.7/h-32.1/h]).^3^ Compared with patients with obstructive sleep apnea at 2590 m (median [IQR], SpO_2_, 85% [83%-88%]; median [IQR] AHI, 86.2/h [67.2/h-103.1/h]),^24^ patients with COPD had lower AHI but similar hypoxemia at 2048 m, probably because of airflow obstruction, ventilation or perfusion mismatch, and diffusion limitation causing mild hypoxemia already at 490 m. In terms of ventilatory control theory, it is interesting to note that the degree of altitude-induced deterioration of breathing stability reflected in the AHI in patients with COPD fell between the modest AHI increase in healthy individuals and the major increase in patients with obstructive sleep apnea syndrome in the cited studies.^3,24^ Presumably, mechanical ventilatory constraints and ventilatory inefficiency that reduces the plant gain of respiratory control in patients with COPD prevented an excessive increase in the AHI to the degree observed in patients with obstructive sleep apnea. Conversely, a relatively enhanced neural respiratory drive in patients with COPD^30^ in the presence of hypoxia-induced chemoreceptor stimulation and altitude-induced pulmonary hypertension promoted greater ventilatory instability compared with healthy individuals.^31,32^ Exploratory analysis revealed that low altitude baseline AHI and nocturnal SpO_2_ were independently associated with high altitude sleep-disordered breathing and nocturnal hypoxemia (eTable 3 and eTable 4 in Supplement 2), suggesting that overnight oximetry at low altitude might serve as a screening tool to identify patients susceptible to sleep disturbed breathing at high altitude.

The reduction of the AHI by NOT was quite pronounced, with even lower values at 2048 m than at 490 m (Table 2). It was associated with an overcorrection of nocturnal SpO_2_ by NOT, greater than values recorded at 490 m (mean [SD], 96% [2%] vs 92% [2%]). Thus, a combined, stabilizing effect of oxygen and of acclimatization on control of breathing might have very effectively reduced the AHI, particularly on the second night at high altitude.

Improvements in sleep structure with NOT were reflected by a higher sleep efficiency, a lower arousal index, a lower percentage of superficial non–rapid eye movement sleep stages 1 and 2, and more rapid eye movement sleep (Table 2). The improvements may have been the result of improved oxygenation and reduced AHI in patients with COPD who received NOT. Subjective sleep quality improved by 9 percentage points after the first night with NOT at 2048 m, similar to the difference of 10 percentage points found to be clinically important in patients with insomnia.^33^ Systolic and diastolic blood pressure at the end of the 6-minute walk test were improved the day after NOT, possibly related to less nocturnal sympathetic activity, as indicated by a lower nocturnal heart rate and a reduced incidence of ventricular extra-beats.

Despite improvements in sleep structure and breathing and better subjective sleep quality with NOT at 2048 m, similar exercise capacity, cognitive performance, and arterial blood gases were observed as with placebo (Table 3). These findings suggest no persistent, measurable effect of NOT on these outcomes.

In addition to the directly measurable effect of NOT on hypoxemia, we speculate that it prevented the hypoxia-induced activation of systemic inflammatory pathways, excessive hyperventilation, and sympathetic overexcitation. This might explain favorable effects of NOT on ARAHE other than just hypoxemia including AMS, COPD exacerbations, and intolerable dyspnea.

### Limitations

This study has limitations. This trial included patients with moderate to severe COPD staying for 2 days at 2048 m; therefore, no extrapolation should be drawn to patients with mild or very severe COPD, higher altitudes, or longer altitude exposures. This study applied 3 L/min NOT through a nasal cannula to improve hypoxemia and sleep-disturbed breathing. This intervention improved SpO_2_ and AHI beyond the values obtained at 490 m, suggesting that 2 L/min NOT might be sufficient during altitude travel in certain patients. The a priori definition of severe hypoxemia (ie, SpO_2 _<75% for >30 minutes at any time during the stay at high altitude), an ARAHE criterion that required study withdrawal and treatment with oxygen, was based on safety concerns. Although severe hypoxemia may not necessarily result in a relevant adverse event when left untreated, in the absence of scientific evidence we considered it as clinically reasonable and ethically well justified not to expose patients with COPD (some of whom also had cardiovascular disease) to more severe and prolonged hypoxia.

## Conclusions

In this randomized, placebo-controlled, crossover trial among lowlanders with moderate to severe COPD ascending for 2 days to 2048 m, we found that altitude exposure was associated with considerable nocturnal hypoxemia as well as breathing and sleep disturbances. The current trial showed that NOT is a highly effective preventive therapy, which not only improved nocturnal SpO_2_, altitude-induced sleep apnea, sleep efficiency, and subjective sleep quality but also prevented ARAHEs. The fact that more than a quarter of the patients with COPD experienced ARAHEs illustrates that a considerable proportion of patients with grade 2 to 3 COPD is not fit to travel to high altitudes without appropriate precautions or preventive NOT.

## Notes

Supplement 1. **Trial Protocol and Statistical Analysis Plan**

Supplement 2. **eAppendix. **Supplementary Methods **eTable 1. **Mixed Linear Regression for the Coprimary Outcome of Mean Nocturnal Oxygen Saturation Including Location, Intervention, and Randomized Exposure Sequence **eTable 2. **Mixed Linear Regression for the Coprimary Outcome of Apnea-Hypopnea Index Including Location, Intervention, and Randomized Exposure Sequence **eTable 3. **Mixed Linear Regression Models of the Apnea-Hypopnea Index at 2048 m Under Placebo Intervention **eTable 4. **Mixed Linear Regression Models of Mean Nocturnal Oxygen Saturation at 2048 m Under Placebo Intervention **eTable 5. **Logistic Regression Analyses of Potential Variables Associated With Altitude-Related Adverse Health Effects (ARAHE at 2048 m) **eReferences.**

Supplement 3. **Data Sharing Statement**
