# Supplementary material for: Effect of Nocturnal Oxygen Therapy on Nocturnal Hypoxemia and Sleep Apnea Among Patients With Chronic Obstructive Pulmonary Disease Traveling to 2048 Meters: A Randomized Clinical Trial
Source: JAMA Netw Open. 2020 Jun 22;3(6):e207940. doi: 10.1001/jamanetworkopen.2020.7940 (PMC7309443; doi:10.1001/jamanetworkopen.2020.7940)
Supplement: Supplement 1. — Trial Protocol and Statistical Analysis Plan [file jamanetwopen-3-e207940-s001.pdf]

1 PATIENTS WITH CHRONIC OBSTRUCTIVE PULMONARY DISEASE (COPD) AT ALTITUDE

Protocol ID: 2013-0088

Version 2.0, Amendment 3

Date 28.4.2014

Principle investigator and Correspondence:
Prof. Dr. med. Konrad E. Bloch
Klinik für Pneumologie
UniversitätsSpital Zürich
RAE-C28
Rämistrasse 100
CH-8091 Zürich
konrad.bloch@usz.ch
Tel: 044 255 3828
Fax: 044 255 4451

Signature:

This protocol has been performed according to the check list of the AGEK. The paragraph numbers refer to corresponding numbers in the check list.

Table of Contents

1 PATIENTS WITH CHRONIC OBSTRUCTIVE PULMONARY DISEASE (COPD) AT ALTITUDE . 1

2 CLINICAL PHARMACEUTICAL TRIAL: BACKGROUND INFORMATION 9

3 OBJECTIVES AND PURPOSE 9

3.1 BACKGROUND, RATIONALE AND PURPOSE OF THE STUDY 9
Chronic obstructive pulmonary disease (COPD) 9
High altitude sojourn and normal physiologic adaptation to altitude 10
Altitude related illness 11
COPD patients: Are they at risk of adverse health effects during an altitude sojourn? 11
Treatment of COPD patients at altitude 12
PH patients: Are they at risk of adverse health effects during an altitude sojourn? 13
Treatment of PH patients at altitude 14
ILD patients: Are they at risk of adverse health effects during an altitude sojourn? 14
Treatment of ILD patients at altitude 14
Study purpose 14
3.2 STUDY RATIONALE AND STUDY POPULATION 15
3.3 HYPOTHESES 15
Study phase 1 15
Study phase 2 16

4 STUDY DESIGN 16

4.1 STUDY OUTCOMES 16
Primary outcome 16
Secondary outcomes 16
4.2 STUDY DESIGN 17
Detailed design for the study phase 1 evaluating the effects of altitude 17
Detailed Design for the study phase 2 evaluating the effect of nocturnal supplemental oxygen 23
Withdrawal criteria 25
Unblinding criteria 25
4.3 INTERVENTIONS TO MINIMISE BIAS 26

5 PARTICIPANT ENTRY 26

5.1 RECRUITMENT 26
5.2 INCLUSION CRITERIA 26
5.3 EXCLUSION CRITERIA 26

6 EVALUATION OF EFFECTS OF ALTITUDE AND OF NOCTURNAL OXYGEN THERAPY 27

6.1 MEASUREMENTS AND TIMING OF MEASUREMENTS 27
Exercise tests 27
Pulmonary function tests 28
Reaction, vigilance and psychomotor performance 28

Specialized cardiovascular and cerebrovascular evaluation 28
Sleep studies 29

7 ASSESSMENT OF SAFETY 30

7.1 SAFETY VARIABLES 30
7.2 FOLLOW-UP OF PATIENTS AFTER ADVERSE EVENTS 30
7.3 ADVERSE EVENTS 30
Adverse Event (AE) 30
Serious Adverse Event (SAE) 31

8 STATISTICS AND DATA ANALYSIS 32

8.1 PRIMARY AND SECONDARY END POINTS (SEE ALSO 4.1.) 32
Primary outcome 32
Secondary outcomes 32
8.2 SAMPLE SIZE ESTIMATION 32
8.3 STATISTICAL ANALYSIS 33
8.4 SIGNIFICANCE LEVEL 33
8.5 HANDLING OF MISSING DATA 33
8.6 DEFINITION OF ANALYSIS PRINCIPLE 33

9 PRECAUTIONS AND DUTIES 33

9.1 SPECIFIC PRECAUTIONS 33

10 DUTIES OF THE INVESTIGATOR 34

10.1 DECLARATION OF CONFORMITY 34
10.2 REPORTING PROCEDURES 34
10.3 INSURANCE 34

11 ETHICAL CONSIDERATIONS 35

11.1 RISK BENEFIT ASSESSMENT 35
11.2 REASON FOR INCLUDING PATIENTS WITH COPD, PD AND ILD 35
11.3 OTHER ETHICAL CONSIDERATIONS 35

12 QUALITY CONTROL 35

12.1 DATA ACCESS, AUDITS, INSPECTIONS AND MONITORING 35
12.2 CONFIDENTIALITY, ARCHIVING AND DESTRUCTION OF TRIAL DOCUMENTS AND PATIENT RECORDS 36
12.3 DESCRIPTION OF DATA IN THE CRFS 36

13 PUBLICATION POLICY 36

14 REFERENCE LIST 36

15 APPENDICES 43

LIST OF INVESTIGATORS AND STUDY SYNOPSIS

**Investigator(s):**	Prof. Dr. med. Konrad E. Bloch<br>Klinik für Pneumologie,<br>UniversitätsSpital Zürich<br>Rämistr. 100, 8091 Zürich<br>Tel.: 044 255 38 28<br>Fax.: 044 255 44 51<br>Email: konrad.bloch@usz.ch<br>Dr. med. Tsogyal Latshang,<br>Klinik für Pneumologie,<br>UniversitätsSpital Zürich<br>Rämistr. 100, 8091 Zürich<br>Tel.: 044 255 38 28<br>Fax.: 044 255 44 51<br>Email: tsogyal.latshang@usz.ch<br><br>PD Dr. Silvia Ulrich<br>UniversitätsSpital Zürich<br>Rämistr. 100, 8091 Zürich<br>Tel.: 044 255 43 62<br>Fax.: 044 255 44 17<br>Email: silvia.ulrich@usz.ch<br><br>Prof. Dr. med. Malcolm Kohler,<br>Klinik für Pneumologie,<br>UniversitätsSpital Zürich<br>Rämistr. 100, 8091 Zürich<br>Tel.: 044 255 38 28<br>Fax.: 044 255 44 51<br>Email: malcolm.kohler@usz.ch<br><br>Michael Furian, BSc<br>Klinik für Pneumologie,<br>UniversitätsSpital Zürich<br>Rämistr. 100, 8091 Zürich<br>Tel.: 044 255 38 28<br>Fax.: 044 255 44 51<br>Email: michael.furian@usz.ch<br><br>Deborah Flück, MMSc<br>Klinik für Pneumologie,<br>UniversitätsSpital Zürich<br>Rämistr. 100, 8091 Zürich<br>Tel.: 044 255 38 28<br>Fax.: 044 255 44 51<br>Email: deborah.flueck@usz.ch

**Study Title:**	Patients with chronic obstructive pulmonary disease (COPD) at altitude
**Protocol Version and Date:**	Protocol ID: 2013-0088<br>Version: 2.0, Amendment 1<br>Date 13.12.2013

**Summary:**	**In study phase 1**, 50 patients with moderate to severe COPD living below 800 m, will be recruited to participate in a randomized cross-over field trial evaluating the hypothesis that exercise capacity during a 4 day sojourn at moderate altitude is reduced in comparison to low altitude. Outcomes will be assessed during 2 days in Zurich (490 m, low altitude baseline), 2 days at Davos Clavadel (1650 m), and 2 days at Davos Jakobshorn (2590 m). The order of altitude exposure will be randomized. The main outcome is the 6 minute walk distance. Additional outcomes are symptom scales, variables from pulmonary function, sleep and vigilance studies, and measures of cardiovascular function. In additional studies 40 patients with precapillary pulmonary hypertension (PH), and 40 patients with interstitial lung disease (ILD) will undergo assessments during 2 days at 490 m, 1 day at 1721 m, and 2 days at 2702 m to evaluate the hypothesis that the 6 minute walk distance is reduced at moderate altitude. If the study locations will not be accessible or will not be appropriate for our needs, the study will take place at different location of comparable altitude.<br>Alternatively, depending on feasibility and logistics, studies will be performed during 2 days in Zurich and during 2 days at only one altitude location, situated between 1650-300 m.<br><br>**In study phase 2**, 50 patients with moderate to severe COPD living below 800 m, will be recruited to participate in randomized, placebo-controlled, double-blind, cross-over trials evaluating the hypothesis that nocturnal supplemental oxygen therapy during a 3-day sojourn at moderate altitude improves exercise performance. After baseline evaluation at 490 m, patients will spend two 3-day periods at altitude (1 day at Samedan, 1721 m, and 2 days at Murtèl Corvatsch, 2702 m) using nocturnal nasal oxygen at a rate of 3 L/Min or placebo oxygen (ambient air at 3 L/Min). The order of treatment modality during the first and second altitude stay will be randomized and treatment periods separated by a 2 week wash-out period at low altitude. The main outcome (6 min walk distance) and the additional outcomes will be the same as in phase 1. Comparisons will be made according to the intention to treat principle among oxygen and placebo oxygen.<br>Alternatively, depending on feasibility and logistics, studies will be performed during 2 days in Zurich and during 2 sojourns of 2 days each at an altitude location situated between 1650-300 m.In additional studies 50 patients with PH, and 50 patients with ILD will be invited to participate in a randomized cross-over trial evaluating the effect of oxygen vs. sham oxygen at 2702m according to a similar protocol as described above for COPD patients. If the study locations will not be accessible or will not be appropriate for our needs, the study will take place at different location of comparable altitude. This protocol has been evaluated by several expert reviewers of the Swiss National Science Foundation and the study will be supported by SNF grant #143875 if approved by the ethics committee.

**Study Duration:**	The first study is scheduled to be has been performed from May-October 2013 (see report in the appendix). Total study duration for each participant was 7 days.<br>The 2nd study is scheduled to be performed from May-October 2014 and 2015. Total study duration for each participant is 9 days.<br>If the study objectives are not achieved by fall 2015, additional measurements will be performed in subsequent years.
**Study Center:**	Klinik für Pneumologie,<br>UniversitätsSpital Zürich<br>Rämistr. 100, 8091 Zürich
**Objective(s):**	Primary study objectives:<br>Study 1<br>To evaluate the effect of short-term exposure to moderate altitude on exercise performance, dyspnea, pulmonary function, cardiovascular function, sleep and ocular motor and vestibular function in patients with COPD, in patients with PH and in patients with ILD.<br>Study 2<br>To evaluate the effect of supplemental oxygen during short-term exposure to moderate altitude on exercise performance, dyspnea, pulmonary function, cardiovascular function, sleep and ocular motor and vestibular function in patients with COPD, in patients with PH and in patients with ILD.
**Number of Subjects:**	Based on the sample size estimation, 50 patients with COPD (GOLD grade 2-3) will be included in each study phase, i.e., 50 in 2013 and 50 in 2014/15. In addition, 50 patients with PH and 50 with ILD will be included.
**Diagnosis and Main Inclusion Criteria:**	- Patients with COPD (GOLD grade 2-3)<br>- Age 18-75 y<br>- Both genders<br>- Informed consent.<br>- Living at low altitude < 800m

**Main Exclusion Criteria:**	- Unstable condition, COPD exacerbation (change in dyspnea, cough or sputum production which may require a change in treatment). Unstable PH (recent requirement for change in treatment), unstable ILD or exacerbated ILD.<br>- Very severe COPD (FEV1 or diffusing capacity <30% predicted, PaO_2 at 490 m <7.3 kPa, $PaCO_2$ at 490 m >6.7 kPa)<br>- Very severe PH, very severe ILD (NYHA 4, diffusing capacity <30% predicted, PaO_2 at 490 m <7.3 kPa)<br>- More than mild or poorly controlled cardiovascular disease<br>- Use of drugs that affect respiratory center drive.<br>- Internal, neurologic or psychiatric disease that interfere with protocol compliance including current heavy smoking (>20 cigarettes per day), inability to perform bicycle exercise.<br>- Previous intolerance to moderate altitude (<2600m)<br>- Pregnancy
**Study Schedule:**	05/2013 through 12/2016
**Statistical Methodology:**	According to principles of cross-over trials "as per protocol" (phase 1) and "intention to treat" phase 2.
**Statement:**	This study will be conducted in compliance with the protocol, the current version of the Declaration of Helsinki, and ICH-GCP as well as all national legal and regulatory requirements. Collection of human biological material will also follow the SAMW Guidelines.

2 CLINICAL PHARMACEUTICAL TRIAL: BACKGROUND INFORMATION

This study is not a clinical pharmaceutical trial.

3 OBJECTIVES AND PURPOSE

3.1 BACKGROUND, RATIONALE AND PURPOSE OF THE STUDY

Chronic obstructive pulmonary disease (COPD)

COPD is characterized by chronic airflow obstruction related to airway inflammation, remodeling and parenchymal destruction of the lung[1]. The pulmonary disease is commonly associated with systemic manifestations such as malnutrition, skeletal muscle dysfunction and co-morbidities including cardiovascular disease, diabetes, osteoporosis, infection, depression and lung cancer. COPD is a major health problem world-wide being one of the leading causes of mortality, morbidity and health care costs. Although COPD occurs in never-smokers, exposure to tobacco smoke is the major risk factor for developing COPD while air pollution from burning biomass fuels and other noxious exposures are increasingly recognized as risk factors. In Switzerland, the prevalence of airflow obstruction has been estimated to be 4-15%, with a higher prevalence in males and in advanced age[2]. Even higher prevalence rates of COPD (up to 20%) are reported in other countries. According to the global initiative for chronic obstructive lung disease criteria (GOLD), the diagnosis of COPD requires a spirometry with a post bronchodilator FEV1/FVC ratio of <0.7 in the appropriate clinical setting; severity of airflow limitation in COPD is classified into 4 grades (GOLD 1-4) according to the value of FEV1 in % predicted[1]. The care of patients with COPD includes reduction of risk factors for progression and complications, in particular smoking cessation, patient education, pharmacological therapy, and supplemental oxygen. Depending on disease severity, inhaled $beta_2$-agonists, anticholinergics and glucocorticosteroids are prescribed and in some cases combined with oral methylxanthins and short-term systemic corticosteroids[1]. Rehabilitation, nutrition counseling and vaccinations are important adjuncts. COPD exacerbations require intensified bronchodilator therapy, systemic glucocorticosteroids[3] and antibiotics and, in certain patients, non-invasive or invasive mechanical ventilation. In selected cases with very severe COPD, lung volume reduction surgery and/or transplantation are performed[4,5].

Pulmonary hypertension

Precapillary pulmonary hypertension (PH) has been defined by the WHO as a mean pulmonary artery pressure $\geq$25mmHg along with a pulmonary capillary occlusion pressure $\leq$15mmHg (to differentiate from pulmonary venous hypertension due to left heart disease).[6] PH is either idiopathic or associated with many different disorders such as collagen vascular disease, portal hypertension, hypoxemic lung diseases, chronic thromboembolism, and many more.[7] The leading symptom in PH is dyspnea on exertion with impaired exercise performance. It can be attributed to: 1st) impaired pulmonary gas exchange due to ventilation-perfusion mismatch, and diffusion impairment due to loss of gas exchange surface, 2nd) inadequate increase in cardiac output during exercise leading to lactic acidosis with increased CO_2 production thereby increasing ventilatory requirements; and 3rd) exercise induced hypoxemia that worsens PH by

hypoxic pulmonary vasoconstriction. With progression of PH, dyspnea occurs on minimal exertion, and syncopes and right heart failure may develop. Although PH is still incurable, recent therapeutic advances, i.e., pulmonary vasodilators and endarterectomy have improved the life expectancy, physical performance and quality of life of PH patients.[8,9]

Interstitial lung disease (ILD)

Interstitial lung diseases emcompass a heterogeneous group of disorders affecting the pulmonary parenchyma by inflammation and fibrosis leading to loss of lung volume.[10] ILD may result from exposure to environmental agents, drugs, connective tissues diseases, or sarcoidosis, but ILD of unknown etiology occurs as well. While idiopathic pulmonary fibrosis is rare (prevalence 1-12/100'000 persons), other ILD such as extrinsic allergic alveolitis are more common (prevalence 50-150/1000 workers in particular occupations). ILD is diagnosed by typical symptoms and signs (i.e., dyspnea, cough, fine inspiratory pulmonary crackles), restrictive ventilatory pattern, impaired gas exchange, and interstitial infiltrates on computed tomographic scans. Depending on the etiology of ILD various therapeutic strategies exists. Possible triggers (environmental agents, drugs) have to be avoided or underlying diseases have to be treated. Different anti-inflammatory, immunosuppressive or antifibrotic agents are used especially in sarcoidosis, hypersensitivity pneumonitis or idiopathic pulmonary fibrosis. Exacerbations are treated with antibiotics, glucocoticoids, and mechanical ventilation.[11] However, some forms of ILD may progress despite therapeutic interventions. In selected cases of very severe ILD lung transplantation is performed.

High altitude sojourn and normal physiologic adaptation to altitude

Worldwide, a large number of low altitude (<800m) residents travel to higher elevations for work and leisure activities. In Switzerland, many holiday resorts are located at moderate altitudes of 1200-2000m, but some lodges and mountain huts are situated at considerably higher altitude (up to 4559 m). Moreover, in America and Asia, some holiday resorts and permanent settlements can be found at altitudes >4000m. In Switzerland and other European countries there is a longstanding tradition of high altitude clinics originally built for the treatment of patients with pulmonary tuberculosis. Some of these institutions, such as the Zürcher Höhenklinik Davos Clavadel (1650m), the Höhenklinik Davos Clavadel (1650m) or the Luzerner Höhenklinik Montana (1473m), have been converted to rehabilitation clinics to which lowlanders are referred for diagnostic evaluation and treatment including for COPD[12-14].

One of the immediate and most important physiologic responses mediated by hypoxic stimulation of peripheral chemoreceptors at altitude is an increase in ventilation. It mitigates hypoxemia but reduces arterial PCO_2 and induces an alkalosis. Once the arterial PCO_2 falls below the apneic threshold, ventilation transiently ceases until the arterial PCO_2 rises again due to metabolic activity[15]. This triggers an overshooting burst of hyperventilation. As a consequence, ventilation waxes and wanes which is called high-altitude periodic breathing. At high altitude periodic breathing may be severe[16], impair sleep[17,18] and even persist during wakefulness. Altitude exposure also affects lung function leading to a reduction in vital capacity which is related in part to reduced respiratory muscle strength[19] and possibly to subclinical interstitial pulmonary edema[20]. Furthermore, hypoxic reflex vasoconstriction of the small pulmonary resistance arteries leads to elevated pulmonary arterial pressure at altitude[21]. Physiological adaptations induced by prolonged altitude exposure are termed acclimatization. Such changes not only involve the respiratory system but virtually all organ systems, including the cardiovascular, renal, digestive and musculoskeletal system and the blood.

Altitude related illness

Traveling to high altitude may affect well-being of unacclimatized lowlanders[22]. Acute Mountain Sickness (AMS), the most common of the altitude related diseases affects 40-50% of lowlanders ascending to moderate altitudes (3000m)[23]. The incidence of AMS depends on ascent rate, sleeping altitude, prior acclimatization and individual susceptibility[24]. AMS generally starts within 8 to 96 hours of arrival at altitude manifesting itself with headache, loss of appetite, weakness, fatigue and insomnia. The latter is related in part to periodic breathing[25]. Severe, untreated AMS may progress to high altitude cerebral edema characterized by ataxia, loss of consciousness and, finally, death. High altitude may also induce excessive pulmonary arterial hypertension and high altitude pulmonary edema (HAPE), a non-cardiogenic pulmonary edema that may cause profound hypoxemia[26]. HAPE is rare at altitudes below 3500m but occurs in about 2-4% of mountaineers at 4559m.

COPD patients: Are they at risk of adverse health effects during an altitude sojourn?

Considering the high prevalence of COPD, the number of patients with the disease is also expected to be high among high altitude sojourners and residents. COPD patients may experience limitations at altitude through several hypothetical mechanisms including ventilatory limitation, impaired gas exchange, alteration in control of breathing, excessively increased pulmonary artery pressure and sympathetic activation, reduced skeletal and respiratory muscle strength, and metabolic and inflammatory responses. However, whether and to which extent these hypothetical mechanisms impact on well-being and physical performance of COPD patients and even increase the risk of adverse events during a stay at altitude has not been appropriately studied.

Some insights have been gained from studies evaluating fitness of COPD patients for air flight. Although commercial aircraft pressurize cabins at cruising altitude, sea level pressure is not maintained for technical and financial reasons. Regulations require that cabin pressure during commercial flights is not lower than 565 mmHg (altitude-equivalent of 8'000 ft., 2438 m) to prevent exposure of passengers to significant hypobaric hypoxia. In healthy subjects during a simulated 20 h long distance flight at minimal cabin pressure corresponding to 2438 m, mean oxygen saturation did not fall below 91%. Most participants reported only minor discomfort and no serious adverse event occurresd[27]. However, 7.4% of participants suffered from acute mountain sickness. In COPD patients, the effects of air travel have been assessed by simulating flight conditions in hypobaric chambers decompressed to an altitude equivalent of 2438 m[28,29], or by letting patients breathe a low inspiratory oxygen fraction of FiO_2=15.1% at ambient pressure near sea level resulting in an inspiratory PO_2 equivalent to an altitude of 2438 m[30-32]. During such brief (<1h) simulations, COPD patients generally felt well although the arterial PO_2 dropped significantly in particular during physical activity resulting in oxygen saturation of <80%[29,31]. Based on various studies >16 equations were derived to predict in-flight PaO_2 from pre-flight measurements of PaO_2, $PaCO_2$, FEV1, diffusing capacity and expected altitude exposure[33-37].

While altitude simulation may help to get some idea about the physiologic response and help to identify patients who might benefit from in-flight oxygen, the clinical relevance of the findings are questionable. No studies have conclusively evaluated whether such tests predict adverse effects during real flights. In fact, although respiratory symptoms seem to be relatively common in COPD patients travelling by airplane (i.e., occur in about 25%[38]) serious events seem to be rare and have not been observed in several studies involving COPD patients during real flights[38-41]. In one questionnaire evaluation including 616 air passengers with lung disease, none of the

patients died during flight[39]. Mortality within 1 month after flight was <1%. Of the 5 patients dying within 1 month after flight, 2 suffered from COPD.

The few studies that have evaluated pulmonary function in COPD patients at altitude do not allow to draw definitive conclusions. Theoretically, airflow resistance at altitude is reduced due to the lower air density. Consistent with this concept, 10 COPD patients at a simulated altitude of 5'500 m in a hypobaric chamber revealed an increase in the FEV1/FVC ratio and in peak expiratory flow rate while vital capacity was slightly decreased[42]. In another study in 18 COPD patients at a much lower simulated altitude of 2438 m, no significant changes in spirometry were recorded[28]. Since patients were breathing 100% oxygen during both studies the results are not representative for natural conditions at altitude. Whether COPD patients experience a reduction in inspiratory muscle force at altitude as observed in healthy subjects[19] has not been reported but might potentially contribute to exercise limitation. A further cause of exercise limitation in COPD patients at altitude may relate to an excessive rise in pulmonary artery pressure due to hypoxic vasoconstriction in the presence of some degree of preexisting pulmonary hypertension although this has not been studied. Excessive sympathetic activation may additionally stress the already impaired cardiovascular system in COPD patients[43].

Observations in COPD patients travelling to real altitude are rare. Karrer and coworkers[44] described blood gas analysis and pulmonary function in newcomers to the Luzerner Höhenklinik Montana, among them 48 with COPD (mean FEV1 56%). Their PaO_2 dropped from 9.36 kPa at Siders (535 m) to 7.94 kPa at Montana (1472 m) which was well tolerated by the patients. In a study by Kelly and coworkers[45], 18 COPD patients (mean FEV1 42% predicted) were transported by car from sea level to Mt. Hutt (2086 m) for a brief sojourn of a few hours. The patient's walk distance was reduced to less than half (from 467 to 245 m). In 8 COPD patients (FEV1 25-78% predicted) travelling from sea level to Mount Washington (1920 m), Vermont, PaO_2 initially dropped from 8.8 to 6.8 kPa but values increased to 7.3 kPa with acclimatization over 4 days; patients performed mild treadmill exercise without notable problems[46]. In the Dutch Asthma Center Davos (1560 m) 37 lowlanders with COPD (FEV1 63% predicted) completed a 5 weeks rehabilitation training which led to an increase in exercise capacity persisting for several months[14]. No other studies on intermediate or long-term sojourn of COPD patients at altitude were identified.

Treatment of COPD patients at altitude

Recent guidelines of the British Thoracic Society suggest that pre-flight assessment of patients with stable respiratory disease should take previous flight experience and time since last exacerbation into consideration[36,37]. In patients at greatest risk such as those with severe COPD (FEV1 <30% predicted) usual care should be optimized and further evaluation by a hypoxic challenge test considered if sea level oxygen saturation is <95%. If PaO_2 or SpO_2 fall below 6.6 kPa or 85%, respectively, during a hypoxic challenge, the use of in-flight oxygen is recommended[36]. Other recommendations have suggested pre-flight hypoxia testing in patients with an FEV1 <1.5 L and in-flight supplemental oxygen if altitude simulation predicts a PaO_2 of 6.7-7.3 kPa[47]. No studies have conclusively evaluated the risks and their prevention in COPD patients during a stay of more than a few hours at altitude. In the absence of scientific data it seems reasonable to encourage patients to refrain from smoking in general and in particular during altitude travel and to continue their usual treatment. Travelers should take along sufficient supply of medication, prednisone and antibiotics to treat exacerbations during altitude sojourns[47]. It has also been suggested that supplemental oxygen should be used to maintain a SaO_2 >90%.

Several studies have demonstrated that this is feasible with various technical aids and forms of application[35,48]. However, it is not known which patients benefit from supplemental oxygen at altitude.
Acetazolamide, a carbonic anhydrase inhibitor, acts as a respiratory stimulant by promoting renal bicarbonate excretion thus correcting the respiratory alkalosis induced by hypoxia. The drug may also stimulate ventilation by carbonic anhydrase inhibition-dependent tissue acidosis within the brain and chemoreceptors. These effects are thought to be responsible for improving the arterial oxygen saturation, reducing periodic breathing and preventing acute mountain sickness in healthy mountaineers[49]. In patients with the obstructive sleep apnea syndrome travelling to altitude we have recently shown that acetazolamide improves oxygen saturation and sleep apnea when given alone or in combination with continuous positive airway pressure (autoCPAP)[50,51]. COPD patients travelling to altitude might also benefit from acetazolamide but there is concern that combined pharmacologic and hypoxic stimulation of ventilation at altitude may exacerbate dyspnea, increase dynamic hyperinflation and even lead to respiratory muscle failure in COPD patients.
Another approach to improve altitude tolerance in COPD patients may be the administration of glucocorticosteroids. Dexamethasone is a potent drug for prevention and treatment of acute mountain sickness. In studies performed at the Capanna Regina Margherita (4559 m) we have shown that dexamethasone reduces the excessive rise in pulmonary artery pressure, improves exercise capacity and prevents high altitude pulmonary edema in susceptible subjects[26,52]. This might be related in part to the beneficial effect of dexamethasone on hypoxia-induced impairment of nitric oxide-mediated arterial relaxation[53] and by modulation of sympathetic activity[54]. Glucocorticosteroids also improve airway inflammation and airflow obstruction in many COPD patients, especially during exacerbations[3]. The downside of glucocorticosteroids is the potential risk of hyperglycemia, fluid retention, elevated blood pressure and sleep disturbances. Osteoporosis, muscle weakness and impairment of immune response are other undesired effects associated with prolonged steroid treatment.
In many COPD patients pulmonary artery pressures is moderately elevated and may rise further due to hypoxic pulmonary vasoconstriction and elevated cardiac output at altitude which may limit right ventricular performance and reduce exercise capacity[55]. Therefore, drugs that reduce pulmonary artery pressure might be of potential use in this setting. In healthy subjects during acute and chronic hypoxia at real or simulated altitude, the phosphodiesterase-5 inhibitor sildenafil reduced elevated pulmonary artery pressures and improved exercise capacity without impairing gas exchange[56,57]. In high altitude pulmonary edema susceptible subjects at 4559 m, we found that the phosphodiesterase-5 inhibitor tadalafil improved oxygen saturation, prevented an excessive rise in pulmonary artery pressure and reduced the incidence of pulmonary edema[26]. In patients with COPD-associated pulmonary hypertension, sildenafil improved pulmonary hemodynamics at rest and during exercise but induced a fall in PaO_2 at rest while gas exchange was not impaired during exercise[58]. Based on the cited studies, COPD patients might benefit from sildenafil or other pulmonary vasodilators during an altitude sojourn but there are concerns about deterioration of gas exchange, headaches and other side effects.

PH patients: Are they at risk of adverse health effects during an altitude sojourn?

Extrapolating from current pathophysiological knowledge one could expect that PH patients may experience excessive hypoxic pulmonary vasoconstriction, endothelial dysfunction, worsened pulmonary hemodynamics, and exaggerated hypoxemia during an altitude sojourn.[59] However, no studies have corroborated this assumption. There have been reports on the occurrence of

HAPE during altitude sojourns in individuals subsequently found to have PH due to various etiologies.[60] Studies during commercial air travel or during flight simulations using normobaric or hypobaric exposure to hypoxia for a few hours revealed that the resulting transient hypoxemia was generally well tolerated by PH patients and that no serious adverse events occurred.[61-63] In conclusion, it is still uncertain whether PH patients are at increased risk of suffering from adverse health effects during altitude travel.

Treatment of PH patients at altitude

Treatments of PH patients during altitude travel have not been specifically tested. However, administration of oxygen in hypoxemic patients in addition to the established PH therapy seems reasonable.[47] Other options include intensifying pre-existing PH target therapy or starting new additional therapies like sustained-release nifedipine, endothelin-receptor antagonists, phosphodiesterase-5 inhibitors or inhaled prostanoids.[9] In the absence of scientific data current guidelines recommend that patients in NYHA functional classes III/IV, and/or PaO2 ≤ 60mmHg (8 kPa) should avoid going to altitudes >1500-2000 m without supplemental oxygen.[64]

ILD patients: Are they at risk of adverse health effects during an altitude sojourn?

As described above for COPD and PH patients, exercise performance of ILD patients at altitude may be reduced because of gas exchange and ventilatory limitation, and excessively increased pulmonary artery pressure. In one study, ILD patients were exposed to normobaric hypoxia simulating commercial airflight.[31] Patients revealed moderate hypoxemia but remained well otherwise. Similarly, in a hypobaric chamber study exposure to a simulated altitude of 2435 min decreased arterial oxygenation of patients with ILD but did not provoque relevant health effects.[65] In a questionnaire survey among airline passengers including 186 with ILD including sarcoidosis no serious adverse effects of commerical air travel was reported.[66] No information of any symptoms was given in this report. There are no conclusive studies that have evaluated altitude tolerance of lowlanders with ILD travelling to altitude.

Treatment of ILD patients at altitude

No conclusive studies on ILD patients traveling to altitude have been performed. Administration of supplemental oxygen seems reasonable in hypoxemic patients.[64] with Another option might be to use drugs that reduce pulmonary hypertension in those with an elevated pulmonary artery pressure already at low alitutde.[9] However, it is not known which patients might benefit from such treatment.

Study purpose

The purpose of this project is to evaluate symptoms, exercise performance, oxygenation, pulmonary function, sleep, breathing disturbances, psychomotor performance and cardiovascular function in COPD patients, and in PH or ILD patients living at low altitude (<800 m) exposed to hypoxia during altitude field studies and to evaluate the effects of supplemental oxygen.

The results of the study are expected to provide a scientific basis for counselling COPD, PH and ILD patients in regard to prevention of undesirable consequences during altitude travel. In addition, the study should provide further insights into the pathophysiology of COPD, PH and ILD during exposure to hypoxia.

3.2 STUDY RATIONALE AND STUDY POPULATION

As outlined above, there is very little scientific data on the effects of altitude on COPD patients, and on PH and ILD patients. Several important questions remain open:

- What is the clinical relevance of the reported reduction in PaO_2 in COPD, PH and ILD patients at moderate altitude; does it affect subjective well-being, and exercise performance?
- What are the effects of an altitude sojourn of a few days on subjective well-being, exercise performance, sleep, cognitive performance and the cardiovascular system? Is there a positive or a negative effect of acclimatization? An increase in the ventilatory sensitivity to hypoxia and hypercapnia during acclimatization may exacerbate dyspnea and dynamic hyperinflation; alternatively, the associated increase in oxygen saturation may improve symptoms and the physical condition.
- Are COPD, PH or ILD patients particularly susceptible to altitude related illness due to their low PaO_2, and consecutive elevation in pulmonary artery pressure?
- How can unfavorable effects of altitude be prevented or treated in COPD, PH and ILD patients?

Since COPD patients commonly travel to high altitude in Switzerland and in many other countries worldwide and since patients are even referred to altitude clinics for treatment, knowledge on the effects of altitude on COPD patients is urgently needed. A similar need exists in regard to PH and ILD patients. The proposed studies will serve as a scientific basis for counseling patients regarding potential adverse effects and treatment options during an altitude sojourn. Based on our experience obtained during several studies from 2007 to 2010[17,50,51,67], and in 2013, we wish to perform the current project at two different locations: The Zürcher Höhenklinik Clavadel (1650 m) and the Jakobshorn Hotel (2590 m) in Davos. The lower altitude (1650 m) is typical for many Alpine resorts in Europe and for altitude rehabilitation clinics. The higher altitude (2590 m) corresponds to that of many Alpine mountain huts and some settlements in America and Asia. Moreover, it is similar to the maximal altitude equivalent that international regulations allow for commercial air flight (i.e., 2438m). Results obtained at the two proposed altitude locations will therefore be widely applicable and clinically highly relevant.
In 2014, the study sites in Davos cannot be used because the Jakobshorn cable car will not be operating. Therefore, we plan to perform altitude studies in the hospital of Samedan (1721m) and at the Corvatsch Murtèl station (2702 m). Both locations provide similar physiologic conditions as those at Davos and the infrastructure is also excellent. For comparison, barometric pressures at Davos Clavadel 1650 m and at Samedan 1721 m are: 623 and 617 Torr, respectively; corresponding values at Jakobshorn 2590 m and Corvatsch Murtèl 2702 m are 554 and 546 Torr, respectively.

3.3 HYPOTHESES

Study phase 1

Lowlanders with COPD (GOLD 2-3) or with PH (NYHA class 2-3) or with ILD (NYHA class 2-3) staying at moderate altitude experience…

1. a reduced exercise performance related to hypoxemia, diffusion limitation, ventilatory limitation and reduced peripheral muscle oxygenation.
2. increased shortness of breath and impaired general subjective well-being.
3. a rise in sympathetic activity, blood pressure and a cerebrovascular dysregulation.

4. nocturnal hypoxemia, sleep related breathing disturbances, impaired psychomotor performance.

Study phase 2

In lowlanders with COPD (GOLD 2-3) or with PH (NYHA class 2-3) or with ILD (NYHA class 2-3) staying at moderate altitude nocturnal supplemental oxygen improves …

1. exercise performance.
2. subjective well-being during daytime.
3. nocturnal oxygen saturation and sleep related breathing disturbances.

4 STUDY DESIGN

4.1 STUDY OUTCOMES

Primary outcome

- Exercise performance (the 6 min walk distance).

Secondary outcomes

- Exercise performance
  - o 6 min walk test: (distance is primary outcome) oxygen saturation and pulse rate at beginning/end
  - o Spiroergometry (1650 m): Maximal work rate, V'O2max; physiologic correlates of exercise limitation, i.e., heart rate, breathing reserve, inspiratory capacity, arterial blood gases
  - o Muscle oxygenation during submaximal exercise (2590 m)
- Subjective well-being
  - o Perceived exertion (Borg CR 10 scale at the end of a 6 min walk; modified MRC dyspnea score)
  - o acute mountain sickness (environmental symptoms cerebral score, AMS-c)
- Pulmonary function
  - o Lung volumes, diffusing capacity, arterial blood gases, maximal respiratory pressures
- Cognitive and psychomotor performance
  - o Psychomotor vigilance reaction time and number of lapses
  - o Trail making time
  - o Vestibular-ocular reflex gain and catch-up saccade pattern
- Cardiovascular and cerebrovascular function
  - o Arterial blood pressure; baroreflex sensitivity; heart rate/heart rate variability
  - o Indices of cerebrovascular auto-regulation and oxygenation measured by Doppler ultrasound and Near-infrared spectroscopy (2590 m only)
  - o Echocardiography: estimated pulmonary artery pressure, left and right atrial and ventricular dimensions and motion
- Sleep study and subjective sleep quality

- Mean and minimal oxygen saturation, oxygen desaturations >3%, transcutaneous PCO_2, prevalence and type of central and obstructive apnea/hypopnea, heart rate
- Subjective sleep quality assessed by a visual analog scale

4.2 STUDY DESIGN

Table 1. Time table of study	
Year	Activity
2013	-months 3-6: preparation of measurement techniques, pilot studies, preparation of field studies<br>-months 3-10: subject recruitment and field studies phase 1<br>-months 10-12: data analysis
2014	-months 1-6: data analysis, preparation of field studies<br>-months 4-10: subject recruitment and field studies phase 2 in COPD patients, and phase 1 in PH and ILD patients<br>-months 10-12: data analysis, publication
2015	-months 1-3: data analysis<br>-months 4-10: complementary measurements if required (completion of field studies phase 2 (COPD, PH, ILD patients, as appropriate)<br>-months 6-12: study completion, data analysis, publication2016
2016	Further complementary measurements if required

This study will be a clinical field study in Zurich and Davos, or in Samedan. The project comprises 2 phases, each consisting in a randomized, cross-over trial performed during several weeks in summer 2013 and 2014-5, respectively. A break between the 1st and the 2nd study phase will allow data analysis so that in the case of unexpected results the protocol can be adjusted.

Detailed design for the study phase 1 evaluating the effects of altitude

The purpose is to investigate effects of a stay at 2 different moderate altitudes on symptoms and physiologic outcomes in COPD patients, and in PH and ILD patients. Examinations will be performed during 2 days at Zurich (490m, low altitude baseline), 2 days at Davos Clavadel and 2 days at Davos Jakobshorn, respectively, in random order (table 2, table 2a, figures 1, 1a & 2, 2a). Transfers between study locations will be by public transportation. The trip Zurich-Davos-Platz is by railroad, from Davos-Platz to Davos Clavadel by bus and from Davos Clavadel to Jakobshorn by bus and cable car. Examinations at the University Hospital of Zurich will take place at the pulmonary function and sleep laboratories of the Pulmonary Division. In Davos, patients will stay in single rooms of the Zürcher Höhenklinik Davos Clavadel and in the Hotel Jakobshorn, respectively. Assessments at the different locations will be similar as detailed in the table 3.

In 2014/15 examinations will be performed during 2 days at Zurich (490m, low altitude baseline), 1 day at the hospital of Samedan (1’721m), and 2 days at the Murtèl station (Corvatsch Mittelstation, 2’702m) (figure 1a & 2). Transfers between study locations will be by public transportation. The trip Zurich-Samedan is by railroad, from Samedan to the cable car base station Surlej by bus, and from Surlej to the Murtèl station (2’702m) by cable car. Examinations at the University Hospital of Zurich will take place at the pulmonary function and sleep

laboratories of the Pulmonary Division. In Samedan, patients will stay in single rooms of the hospital Samedan and in the hotel at Murtèl, respectively. Assessments are listed in the table 3.

Alternatively, depending on feasibility and logistics, examinations will be performed during 2 days at Zurich (490m, low altitude baseline), and during 2 days at only one altitude location situated at a moderate altitude of 1650-3000 m. For example, the hotel Salastrains, situated at 2044 m above St. Moritz, would be a suitable location. All arrangements would be similar to those described above for the protocol with 2 different altitude locations.

Table 2. Randomized sequences of altitude exposure in study phase 1			
Day -60 to 0: Subject screening, recruitment, randomization			
Sequence A	Sequence B	Sequence C	Sequence D
490 m, ZH 1-2	490 m, ZH 1-2	1650m, CL 1-2	2590 m, JH 1-2
1650 m, CL 1-2	2590 m, JH 1-2	2590 m, JH 1-2	1650 m, CL 1-2
2590 m, JH 1-2	1650 m, CL 1-2	490 m, ZH 1-2	490 m, ZH 1-2

Table 2a. Randomized sequences of altitude exposure in study phase 1 (PH and ILD patients)	
Day -60 to 0: Subject screening, recruitment, randomization	
Sequence A	Sequence B
490 m, Zurich 1-2	1721 m, Samedan 1
1721 m, Samedan 1	2702 m, Murtèl 1-2
2702 m, Murtèl 1-2	490 m, Zurich 1-2

Table 2b. Randomized sequences of altitude exposure in study phase 1 (PH and ILD patients) if only one altitude location is used	
Day -60 to 0: Subject screening, recruitment, randomization	
Sequence A	Sequence B
490 m, Zurich 1-2	Altitude location (at 1650-3000 m) 1-2
Altitude location (at 1650-3000 m) 1-2	490 m, Zurich 1-2

The design will have 2 sequences similar to those in study phase 2.

The study design of phase 1 is displayed in figure 1 below.

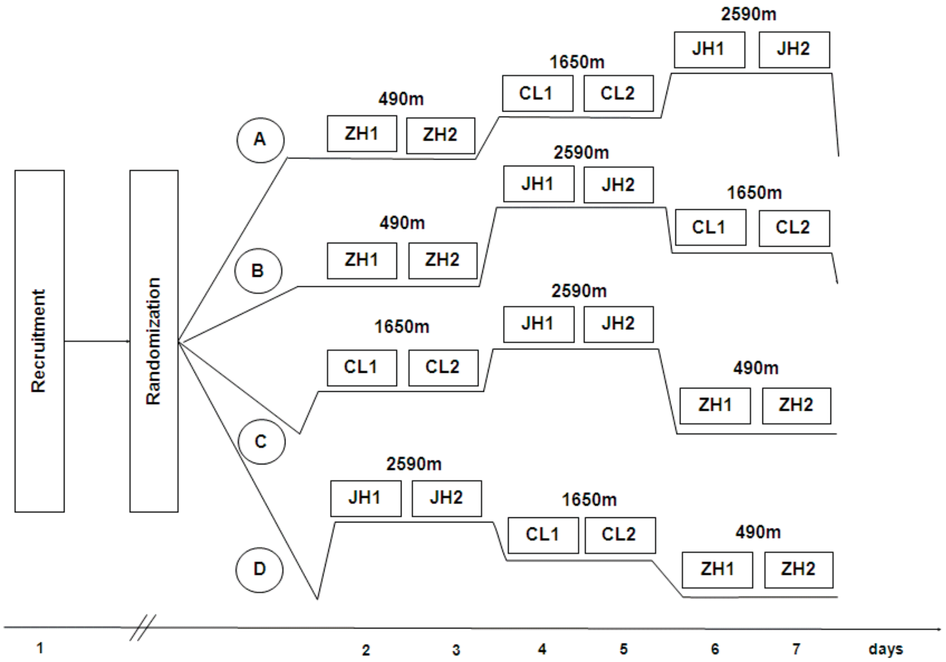

Figure 1: Study design, phase 1: After recruitment, patients will be randomized to one of four sequences of altitude exposure (A to D); ZH 1, ZH 2 = Baseline examinations at 490 m; CL1, CL2= altitude examinations at 1650m; JH1, JH2 = altitude examinations at 2590m.

The study design of phase 1 for PH and ILD patients is displayed in figure 1a below.

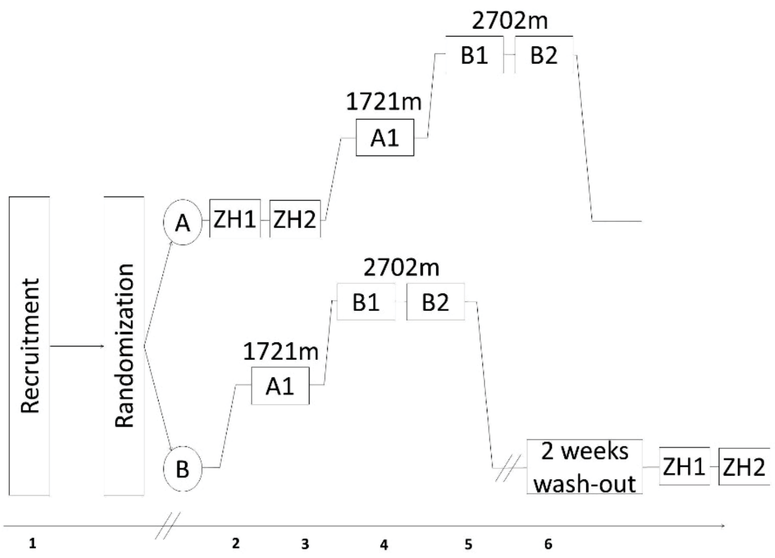

Figure 1a: Study design, phase 1: After recruitment, patients will be randomized to one of 2 sequences of altitude exposure, A or B; ZH 1, ZH 2 = Baseline examinations at 490 m; A1 = altitude examinations at 1721m; B1, B2 = altitude examinations at 2702m. If only one altitude location is used, the intermediate step (A1) falls away and B1 and B2 will correspond to examinations at an altitude of 1650-3000 m, depending on feasibility and logistics.

Number of examination day	1	2	3	4	5	6	7
Visits*	**Recr.**	**ZH1**	**ZH2**	**CL1**	**CL2**	**JH1**	**JH2**
Informed consent							
In-/exclusion criteria							
Demographic data, past medical history							
Lung function test, aBGA**,<br>24h ambulatory pulse oximetry							
Medical history, clinical examination, questionnaires							
Polysomnography							
Reaction tests							
Echocardiography							
6 minute walk test							
Lung function test							
NIRS*** und transcranial Doppler							
Spiroergometry							

*Recr. = Recruitment day; ZH1, ZH2 = Zurich, 490 m; CL1, CL2 = Davos Clavadel, 1650 m; JH1, JH2 = Davos Jakobshorn, 2590 m. 1 and 2 denotes day 1 and day 2 at corresponding altitude. **aBGA = arterial blood gas analysis. ***NIRS = near infra-red spectroscopy.
Hatched field represent days without corresponding examination

Figure 2: Overview of all examinations at each location in study phase 1. If only one altitude location is used, the studies JH1 and JH2 will be performed at a moderate altitude of 1650-3000 m, and the studies CL1 and CL2 fall away.

Number of examination day	1	2	3	4	5	**6**
Visits*	**Recr.**	**ZH1**	**ZH2**	**CL1**	**JH1**	**JH2**
Informed consent						
In-/exclusion criteria						
Demographic data, past medical history						
Lung function test, aBGA**, 24h ambulatory pulse oximetry						
Medical history, clinical examination, questionnaires						
Polysomnography						
Reaction tests						
Echocardiography						
6 minute walk test						
Lung function test						
NIRS*** und transcranial Doppler						
Spiroergometry						

*Recr. = Recruitment day; ZH1, ZH2 = Zurich, 490 m; CL1= hospital Samedan, 1721 m; JH1, JH2 =, Murtèl, Corvatsch 2702 m. 1 and 2 denotes day 1 and day 2 at corresponding altitude. **aBGA = arterial blood gas analysis. ***NIRS = near infra-red spectroscopy.
Hatched field represent days without corresponding examination

Figure 2a: Overview of all examinations at each location in study phase 1 for PH and ILD patients. If only one altitude location is used, the studies at C1 fall away and studies JH1 and JH2 will be performed at a moderate altitude of 1650-3000 m.

Table 3. Schedule of assessments*		
Day 1	16:30	Arrival
	16:45	Questionnaires, clinical examination
	18:30	Dinner and preparation for sleep study
Night 1	22:00	Sleep study
Day 2	06:00	Wake up; clinical examination, baroreceptor sensitivity
	08:00	Breakfast
	09:00	Questionnaires, psychomotor vigilance tests (psychomotor vigilance test, trail making test, vestibular-ocular test)
	10:00	Break
	10:30	Pulmonary function
	11:30	6 min walk test
	12:00	Lunch
	13:00	Leisure time
	14:30	Echocardiography
	15:30	1650 m: spiroergometry; 2590 m: muscle oxygenation and cerebrovascular response
	16:30	Break
	17:00	Questionnaires, clinical examination
	18:30	Dinner and preparation for sleep study
Night 2	22:00	Sleep study
Day 3	06:00	Wake up; clinical examination, baroreceptor sensitivity
	08:00	Breakfast
	09:00	Questionnaires, psychomotor vigilance tests (psychomotor vigilance test, trail making test, vestibular-ocular test)
	10:00	Break
	10:30	Pulmonary function
	11:30	6 min walk test
	12:30	Lunch; then departure

* Assessments will be similar at all study locations with the following exceptions:
Samedan, 1721m: physiologic evaluation will include spirometry, body plethysmography, diffusing capacity, sniff nasal pressure, maximal exercise (spiroergometry)
Murtèl Corvatsch, 2702 m: as at 1721 m but without body plethysmography and spiroergometry; instead, measurements of cerebrovascular blood flow and muscle oxygen at rest and during submaximal exercise.

Detailed Design for the study phase 2 evaluating the effect of nocturnal supplemental oxygen

This will be a randomized, sham-controlled, double-blind, cross-over trial evaluating the effects of nocturnal supplemental oxygen at altitude (table 4, figure 3 & 4). After 2 baseline examinations in Zurich patients will spend 3 days at altitude (1 day in Samedan, 2 days at Murtèl, Corvatsch) and then return home for a 2-weeks washout period. Subsequently, they will spend a 2nd 3-day period at altitude. Patients will receive nocturnal supplemental oxygen (3 L/min per nasal cannula) from a concentrator validated for use at altitude (EverFlow, Philips Respironics) during the 1st altitude stay and room air (sham oxygen) from an identically looking, modified concentrator during the 2nd altitude stay, or vice-versa according to randomization. Assessments will be as outlined in table 3 for study phase 1 with the exception that patients will transfer from Samedan to Corvatsch Murtèl already in the afternoon of day 2 (table 4, below).

Table 4. Sequences of interventions in study phase 2	
Day -60 to 0: Subject screening, recruitment, randomization	
Sequence A	Sequence B
490 m, ZH 1-2	490 m, ZH 1-2
Oxygen: 1721 m, Samedan 1	Sham: 1721 m, Samedan 1
Oxygen: 2590 m, Corvatsch Murtèl 1-2	Sham: 2702 m, Corvatsch Murtèl 1-2
Wash-out phase	Wash–out phase
Sham: 1721 m, Samedan 2	Oxygen: 1721 m, Samedan 2
Sham: 2702 m, Corvatsch Murtèl 3-4	Oxygen: 2702 m, Corvatsch Murtèl 3-4

Table 4a. Sequences of interventions in study phase 2 if only one altitude location is used	
Day -60 to 0: Subject screening, recruitment, randomization	
Sequence A	Sequence B
490 m, ZH 1-2	490 m, ZH 1-2
Oxygen: altitude location at 1650-3000 m	Sham: altitude location at 1650-3000 m
Wash-out phase	Wash–out phase
Sham: altitude location at 1650-3000 m	Oxygen: altitude location at 1650-3000 m

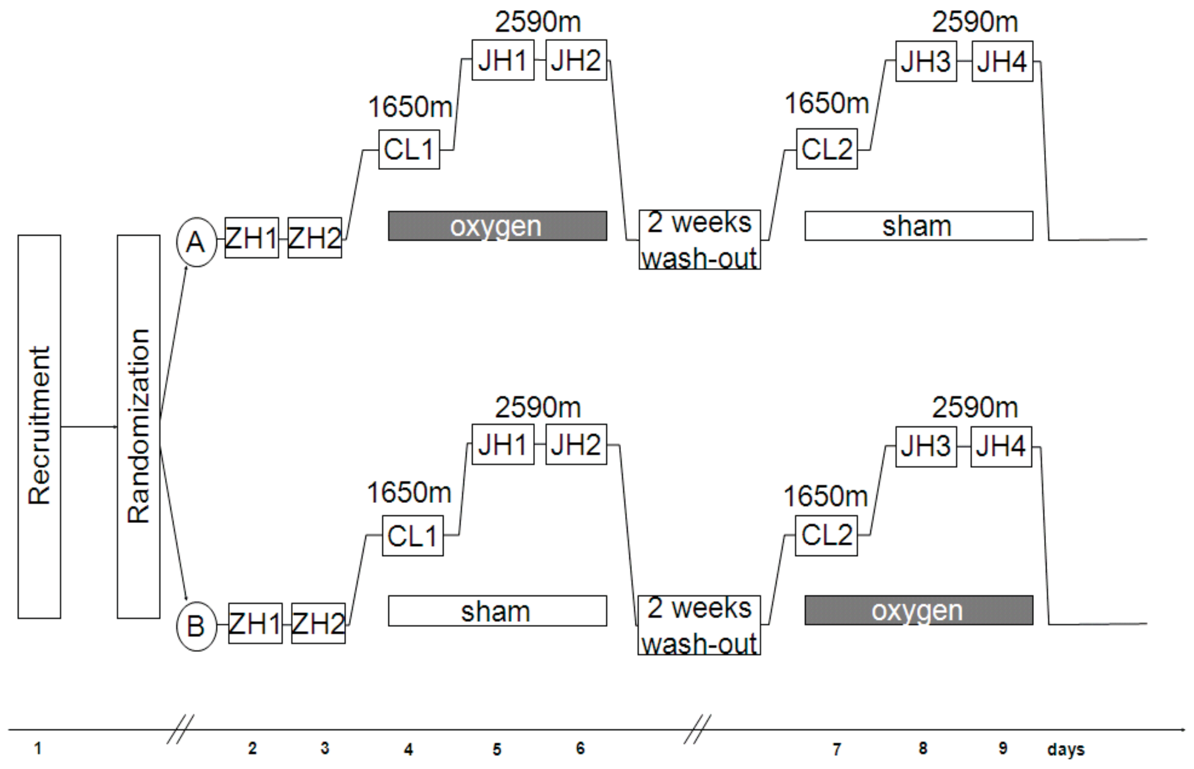

Figure 3: Baseline examinations will be performed during 2 days at ZH1, ZH2 at 490m. Randomized trial of nocturnal oxygen during two 3-day periods at altitude (1st period: CL1(Samedan 1): day 4 at 1721m; JH1, JH2 (Corvatsch Murtèl 1-2): day 5 and 6 at 2702m; 2nd period CL2 (Samedan 2): day 7 at 1721m; JH3, JH4 (Corvatsch Murtèl 3-4): day 8 and 9 at 2590 m). Patients randomized to sequence A will receive nocturnal oxygen during the 3 days at altitude. After a 2-weeks wash-out period at <800 m, they will spend the 2nd 3-day period at altitude receiving sham oxygen. The sequence of oxygen/sham is reversed in patients randomized to sequence B. The study will end after the 2nd altitude sojourn. If only one altitude location is used, the intermediate day in each study phase (CL1 and CL2) falls away. The studies at JH1 and JH2 will be performed at a moderate altitude of 1650-3000 m, depending on feasibility and logistics.

Number of examination day	1	2	3	4	5	6	7	8	9
Visits*	**Recr.**	**ZH1**	**ZH2**	**CL1**	**JH1**	**JH2**	**CL2**	**JH3**	**JH4**
Informed consent									
In-/exclusion criteria									
Demographic data, past medical history									
Lung function test, aBGA**, 24h ambulatory pulse oximetry									
Medical history, clinical examination, questionnaires									
Polysomnography									
Reaction tests									
Echocardiography									
6 min walk test									
Lung function test									
NIRS*** und Transcranial Doppler									
Spiroergometry									

*Recr. = Recruitment day. ZH1, ZH2 = Zurich, 490m; CL1, CL2 = Samedan, 1721m; JH1, JH2=, Corvatsch Murtèl 2702 m, 2 and 3 denotes day 2 and day 3 at corresponding altitude. **aBGA= arterial blood gas analysis. ***NIRS = near infra-red spectroscopy.

Figure 4: Overview of all examinations at each location in study phase 2. If only one altitude location is used, the studies CL1 and CL2 fall away, and studies at JH1-JH2 and JH3-JH4 will be performed at a moderate altitude of 1650-3000 m.

Withdrawal criteria

The following criteria will be considered as indication for withdrawing a patient from the study if treatment with supplemental oxygen or appropriate medical treatment is not readily improving the condition: significant acute mountain sickness (AMS-c score >2); dyspnea at rest; chest pain at rest; systolic blood pressure >200 mmHg, diastolic blood pressure >110 mmHg; SpO_2 <80% at rest or <75% during exercise.

Unblinding criteria

Investigators will be unblinded only after analyses of all data. Exceptions are serious adverse events or other conditions that require immediate knowledge on oxygen or sham treatment to allow prevention of adverse health effects to the patient.

4.3 INTERVENTIONS TO MINIMISE BIAS

The study is designed according to a randomized cross-over trial balanced for the ascent protocol during study phase 1 and to the order of the oxygen and placebo during study phase 2.
Randomization will be performed by letting patients draw an envelope from a set containing a code defining the order of interventions. Envelops will be prepared in a way that assures a balanced design in regard to oxygen/sham and pulsed/continuous oxygen/sham. All patients and investigators are blinded for oxygen/sham or vice versa during study phase 2 by the use of identically looking concentrators producing room air and oxygen.

5 PARTICIPANT ENTRY

5.1 RECRUITMENT

Patients diagnosed with stable COPD, PH or ILD will be asked to participate. They will be recruited among patients attending the Pulmonary Division University Hospital of Zurich and by advertisements distributed to the Zurich Lung League “Lunge Zürich” and newspapers.
During pre-study screening (recruitment examination), a detailed medical history and review of medical records will be performed to identify any risk factors that may predispose to adverse altitude effects (see exclusion criteria). Pulmonary function, arterial blood gases, resting ECG and a 24 h ambulatory pulse oximetry at the patient’s home will be obtained.

5.2 INCLUSION CRITERIA

- COPD diagnosed according to GOLD criteria, grades 2-3.
- Precapillary PH, diagnosed according to international guidelines, NYHA class 2-3.
- Interstitial lung disease diagnosed according to international guidelines, NYHA class 2-3.
- Age 18 to 75 years, both genders.
- Residence at low altitude (<800m).
- Informed consent.

5.3 EXCLUSION CRITERIA

- Unstable condition and COPD exacerbation defined as a change in the dyspnea, cough or sputum production beyond normal day-to-day variation, acute in onset and which may require a change in treatment; a change in baseline medication within the last 30 days.
- Very severe COPD (i.e. GOLD 4, FEV1<30% predicted), severe gas exchange or hemodynamic impairment: diffusing capacity <30% predicted; hypoxemia requiring oxygen therapy at sea level (PaO_2<7.3 kPa); hypoventilation ($PaCO_2$ 6.7> kPa); mean pulmonary artery pressure measured by right heart catheter >40 mmHg or pulmonary artery systolic pressure estimated by echocardiography >50 mmHg.
- Patients with very severe PH, NYHA class 4, unstable PH defined by a change of specific drug therapy within the last 30 days, hypoxemia requiring oxygen therapy at sea level (PaO_2<7.3 kPa); at 490m.
- Patients with very advanced ILD, NYHA class 4, unstable or exacerbated ILD, hypoxemia requiring oxygen therapy at sea level (PaO_2<7.3 kPa).

- More than mild or inadequately controlled cardiovascular disease such as uncontrolled systemic arterial hypertension, coronary artery disease, previous stroke; obstructive sleep apnea syndrome (overlap syndrome); pneumothorax in the last 2 months.
- Use of drugs that affect respiratory center drive (sedatives or sleep inducing drugs, morphine or codeine derivates).
- Internal, neurologic or psychiatric disease that interfere with protocol compliance including current heavy smoking (>20 cigarettes per day), inability to perform bicycle exercise.
- Previous intolerance to moderate altitude (<2600m).
- Exposure to altitudes >1500m for >2 days within the last 4 weeks before the study.Pregnant patients (women potentially capable of childbearing, i.e., who are not yet menopausal and had their last menstrual period less than 12 months ago, who had no hysterectomy,ovarectomy, or surgery to occlude the fallopian tubes, will be tested for pregnancy, on site, at the beginning of the study, by urinary β- HCG-concentration (lifeSign Status hCG Serum/Urine). The urine sample will not be stored.)
- Nursing patients.

6 EVALUATION OF EFFECTS OF ALTITUDE AND OF NOCTURNAL OXYGEN THERAPY

6.1 MEASUREMENTS AND TIMING OF MEASUREMENTS

The timing of measurements described below is outlined in table 3 (4.2. above).

Exercise tests

The 6 min walk test and bicycle exercise are performed according to published standards[68,69].
During the 6 min walk test, patients will be monitored by a portable pulse oximeter and fill in a 10 point Borg scale at the end of the test. Walk distance, SpO_2 and pulse rate at beginning and end of the test will be recorded.
Maximal bicycle exercise will be performed with a progressive ramp protocol to exhaustion. Patients will indicate the perceived exertion on the Borg CR10 scale. Ventilation, gas exchange, oxygen saturation and the ECG will be recorded (ZAN, Oberthulba, Germany). A radial artery blood sample will be drawn at rest and during maximal exercise and analyzed on site immediately by a blood-gas analyser (RAPIDPOINT 405, Siemens, Bad Naunheim, GE). Data will be stored in anonymized format using a unique participant number. No blood will be stored for further analysis.

Symptom evaluation

A complete medical history will be obtained including previous altitude exposure and tolerance, altitude related illness. Physical examination will include weight, height, blood pressure, pulse rate, oxygen saturation by pulse oximetry, cardiac and pulmonary auscultation, evaluation of ataxia by the heel walking test. The COPD assessment test (CAT) will be performed[70]. Perceived exertion will be assessed by the Borg CR10 scale[71], dyspnea by the modified Medical Research Council dyspnea score[72], and acute mountain sickness by the environmental symptoms questionnaire cerebral subscore (AMS-c)[73]. Sleepiness will be assessed by the Epworth sleepiness scale (ESS)[74] and the Stanford sleepiness scale[75].

Pulmonary function tests

Spirometry, lung volumes, diffusing capacity, maximal inspiratory, expiratory and sniff nasal pressures are measured according to published standards (Vmax, SensorMedics) [76,77]. Ambulatory nocturnal pulse oximetry will be performed with a miniaturized device (Minolta 300i, Konica Minolta Sensing Inc.) placed at the wrist and a finger clip. Arterial oxygen saturation is recorded over the course of a night during which the patient sleeps at home. Data are downloaded in the next morning and the mean nocturnal oxygen saturation and the number of dips >3% related to the time the patient spent in bed will be determined.

Reaction, vigilance and psychomotor performance

The tests as described below provide multiple, well defined parameters of psychomotor performance. They will be performed in a quiet room with subjects wearing sound protective ear covers. The light in the room will be dimmed.

Psychomotor Vigilance Test (PVT): Vigilance will be assessed by the Psychomotor Vigilance Test (PVT)[78] which is sensitive for effects of sleep restriction/disturbance and hypoxia. As described by Dinges et al the PVT measures the reaction time to a visual stimulus (LED) presented in variable intervals between 1 to 10 s over a period of 10-15 min. The subjects respond by pressing a button on a hand-held device (Stowood Scientific Instruments Ltd., Oxford).

Trail making test A: This Trail making test A[79] provides information about executive functions and mental flexibility. On a paper sheet are numbers from 1 to 90. Before starting the test, the patient has to draw 1 of 4 sheets with differently randomized numbers. The patient has to connect the numbers from 1 to 90 by drawing a line from number to number. The next higher number is always neighboring the previous number.

Vestibular-ocular reflex: The vestibular-ocular reflex stabilizes gaze during head rotations with equal eye rotations to the opposite direction. Hypoxia will impair this reflex causing balance problems and oscillopsia (jiggling vision) in more severe cases. This will lead to fast correction movements of the eyes to refocus the target (catch-up saccades). The video head impulse test is performed in the sitting patient focusing on a target straight ahead. His head will be turned in approximately 20 small, unpredictable movements to both sides by the examiner (10-15°, 150-200°/s)[80,81]. The patient wears a light video-goggle (ICS Impulse, GN Otometrics, Denmark) which will record the movements of his head and his eyes[82,83]. The gain of the vestibulo-ocular reflex as well as timing and amplitude of the catch-up saccades will be analyzed. [840,85],[86,87] Saccade metrics including amplitude, latency and timing are sensitive markers in response to hypoxia. In addition, the ability to generate anti-saccades will likely be impaired.

Specialized cardiovascular and cerebrovascular evaluation

Echocardiography: Patients will be bedded on a couch and allowed to relax for at least 15 minutes. Cardiac morphological and functional parameters will be assessed by comprehensive two-dimensional doppler echocardiography. Echocardiography data will be stored and analyzed by an investigator blinded to the patients' clinical data.[88] Doppler imaging of the tricuspid annulus will be assessed according to standard methods.[89] This will allow us to assess the following parameters: systolic pulmonary artery pressure (RVsys, calculated from the tricuspid regurgitation velocity by using the modified Bernoulli equation: RVsys =4x(vmax)4 where vmax is the maximum of the regurgitation velocity jet measured over the tricuspid valve); the right and left atrial and ventricular fractional area change (%) and the tricuspid annular plain systolic excursion (mm).[89]

Cardiovascular and cerebrovascular control: Measurements will be performed during a 30 min quiet rest period and during two 6 min intervals of exercise, separated by a 6 min period of rest. Submaximal constant load exercise at 50% of predicted maximal heart rate will be performed using a couch cycle-ergometer with 45% semi-recumbent position (Ergoline Ergometrics 900, Schiller AG, Switzerland) with the head stabilized by a deflatable pillow. In the studies using nocturnal oxygen supplementation (2nd part of project), the load will be kept identical during the active and sham treatment period. Resting heart rate and its variability in the high and low frequency band reflecting sympatho-vagal activation the *baroreceptor reflex* will be assessed by continuous measurement of the ECG and continuous beat by beat blood pressure by the Finapres® technique[90]. *Quadriceps muscle tissue oxygen* will be monitored by near infrared spectroscopy (NIRO 200 NX, Hamamatsu, Shizuoka, Japan) along with peripheral pulse oximetry. In healthy volunteers, skeletal muscle deoxygenation occurs more rapidly at altitude [91], and it is expected that this decrease may be even more pronounced in COPD patients, thereby contributing to early onset of fatigue at altitude. Thus, the hypothesis will be tested that, for a given cardiac output, COPD patients will desaturate to a higher degree at 2590 m vs. 490 m. *Cerebrovascular blood flow*: Patients will be fitted with a mouthpiece and nose clip. Respired gases will be sampled continuously. End-tidal (i.e., arterial) PO_2 and PCO_2 will be measured by gas analyzers using the technique of dynamic end-tidal forcing [92,93]. The right middle cerebral artery (MCA) blood flow velocity as an index of cerebral blood flow will be measured using transcranial Doppler ultrasound (TC22, SciMed, Bristol, England) [92-94]. Measurements will include the maximum (VP) and intensity-weighted mean Doppler frequency shifts and the power of the Doppler signal, an index of the cross-sectional area of the vessel. Without a change in power (i.e. at a constant vessel cross-sectional area), VP is considered a reliable index of global cerebral flow. Changes in cerebral vascular tone will be calculated as cerebrovascular conductance, which is the change in VP for a given change in mean arterial pressure [95]. Recent studies have suggested that cerebral autoregulation is impaired in COPD patients during exercise. Since cerebral autoregulation is disturbed in healthy subjects exposed to hypoxia at altitude we hypothesize that COPD patients exercising at 2590 m will reveal cerebrovascular dysfunction compared to 490 m that might be modified by oxygen administration.

Sleep studies

Nocturnal polysomnography will be performed according to standard techniques as described previously. In addition to central EEG derivations, EOG and submental EMG, measurements include calibrated respiratory inductive plethysmography (RespiratracePT, Nims, Miami Beach, USA), pulse oximetry, end-tidal (CapnoCheck Plus, ResMed, Basel Switzerland) and transcutaneous PCO_2 (Tosca, Radiometer, Basel, Switzerland), pulse transit time, nasal pressure swings[96,97], oral thermistor, NIRS of cerebral tissue oxygenation from 2 miniaturized frontal sensors, bilateral sub costal surface EMG of the diaphragm to assist in differentiation of obstructive from central apnea/hypopnea [98]. Sleep and arousals will be scored according to standard criteria as described by Rechtschaffen und Kales and by the American Academy of Sleep Medicine guidelines. An apnea or hypopnea will be scored if the amplitude of the nasal pressure swings or the sum signal of the respiratory inductive plethysmography falls below 50% of baseline during the previous 2 minutes for >10 seconds[67]. Obstructive will be differentiated from central events based on rib cage-abdominal asynchronous and paradoxical motion, deformations of the nasal pressure curve suggesting inspiratory flow limitation, and diaphragmatic surface EMG[98]. The apnea/hypopnea and oxygen desaturation index (SpO_2 dips >3%) will be computed as mean number of events/h.

7 ASSESSMENT OF SAFETY

7.1 SAFETY VARIABLES

Adverse events or reactions will be evaluated by daily physical examinations, evaluation of questionnaires, through the acquisition of the variables described above at the time points listed in table 3 and through voluntary report at any time during the study.

7.2 FOLLOW-UP OF PATIENTS AFTER ADVERSE EVENTS

Patients will be followed until the event resolves or until 12 weeks after the event is reported.

7.3 ADVERSE EVENTS

Adverse Event (AE)

Per the International Conference of Harmonisation (ICH), an AE is any untoward medical occurrence in a patient or clinical investigation subject underwent an interventional procedure, which does not necessarily have a causal relationship with the treatment. An AE can therefore be any unfavourable and unintended sign (including an abnormal laboratory finding), symptom, or disease temporally associated with the use of a medicinal product, whether or not considered related to the study-intervention. Pre-existing conditions which worsen during a study are to be reported as AEs. Each participant will be examined according to checklists and with questionnaires during the study. Adverse events will be recorded in prepared forms and checklists (see CRF). Adverse events that occur intermittently should be recorded as one AE. Adverse events should be collected and reported for up to 14 days after the study intervention.

Intensity: All clinical AEs encountered during the clinical study will be reported on the AE page of the CRF. Intensity of AEs will be graded on a four-points scale (mild, moderate, severe, life-threatening) and reported in detail in the CRF.

Mild – Discomfort noticed but no disruption of normal daily activity.
Moderate – Discomfort sufficient to reduce or affect daily activity.
Severe – Inability to work or perform normal daily activity.
Life threatening – Represents an immediate threat to life.

Study intervention – Adverse Event Relationship: Relationship of the AE on the treatment should always be assessed by the investigator. AE categories for determining relationship to altitude:

PROBABLE (must have first three)

This category applies to those AEs which are considered, with a high degree of certainty, to be related to the altitude. An AE may be considered probable, if:

1. It follows a reasonable temporal sequence from altitude.
2. It cannot be reasonably explained by the known characteristics of the subject’s clinical state or environmental administered to the subject.
3. It follows a known pattern of response to the suspected intervention.

POSSIBLE (must have first two)

This category applies to those AEs in which the connection with the altitude appears unlikely but cannot be ruled out with certainty. An AE may be considered possible if, or when:

1. It follows a reasonable temporal sequence from altitude.
2. It may have been produced by the subject’s clinical state or environmental factors administered to the subject.

3. It follows a known pattern of response to the suspected intervention

REMOTE (must have first two)

In general, this category is applicable to an AE which meets the following criteria:
1. It does not follow a reasonable temporal sequence from altitude.
2. It may readily have been produced by the subject's clinical state or environmental factors administered to the subject.
3. It does not follow a known pattern of response to the suspected intervention.

Serious Adverse Event (SAE)

Any untoward and unexpected medical occurrence or effect that:

- Results in death,
- Is life-threatening – refers to an event in which the subject was at risk of death at the time of the event; it does not refer to an event which hypothetically might have caused death if it were more severe,
- Requires hospitalisation, or prolongation of existing inpatients' hospitalisation,
- Results in persistent or significant disability or incapacity,
- Is a congenital anomaly or birth defect.
- Medical judgement should be exercised in deciding whether an AE is serious in other situations. Important AEs that are not immediately life-threatening or do not result in death or hospitalisation but may jeopardise the subject or may require intervention to prevent one of the other outcomes listed in the definition above, should also be considered serious.

SAEs will be immediately reported to the Ethics committee of the Canton Zürich. A special form will be completed.

An emergency action plan will be prepared with instructions on first aid and evacuation. In case of an emergency at Davos Jakobshorn the patient will receive first aid and treatment and will be evacuated to the hospital of Davos if necessary. Descent by cable car is available for emergencies also at night times. First aid materials will be readily available at all study locations. Light forms of Acute Mountain Sickness with symptoms such as headache, light-headedness, fatigue and insomnia may occur. More severe symptoms are unlikely because of the moderate altitude. Nevertheless, oxygen administration to reverse hypoxia and subsequent descent to lower altitude may be performed in case of emergencies. Skin irritation may appear due to attachments of the sensors. Arterial blood may cause minor pain and hematoma at the puncture site may occur. A first aid kit with medication for treatment of altitude related illness and for skin irritation and hematoma from puncture will be readily available.

8 Statistics and Data Analysis

8.1 Primary and secondary end points (see also 4.1.)

Primary outcome

Exercise performance (6 min walk distance).

Secondary outcomes

- Exercise performance
  - 6 min walk test: (distance is primary outcome) oxygen saturation and pulse rate at beginning/ end
  - Spiroergometry (1650 m): Maximal work rate, V'O2max; physiologic correlates of exercise limitation, i.e., heart rate, breathing reserve, inspiratory capacity, arterial blood gases
  - Muscle oxygenation during submaximal exercise (2590 m)
- Subjective well-being
  - Perceived exertion (Borg CR 10 scale at the end of a 6 min walk; modified MRC dyspnea score)
  - acute mountain sickness (environmental symptoms cerebral score, AMS-c)
- Pulmonary function
  - Lung volumes, diffusing capacity, arterial blood gases, maximal respiratory pressures
- Reaction and psychomotor performance
  - Psychomotor vigilance reaction time and number of lapses
  - Trail making time
  - Ocular saccade pattern
- Cardiovascular and cerebrovascular function
  - Arterial blood pressure; baroreflex sensitivity; heart rate/heart rate variability
  - Indices of cerebrovascular auto-regulation and oxygenation measured by Doppler ultrasound and Near-infrared spectroscopy (2590 m only)
  - Echocardiography: estimated pulmonary artery pressure, left and right atrial and ventricular dimensions and motion
- Sleep study and subjective sleep quality
  - Mean and minimal oxygen saturation, oxygen desaturations >3%, transcutaneous PCO_2, prevalence and type of central and obstructive apnea/hypopnea, heart rate
  - Subjective sleep quality assessed by a visual analog scale

8.2 Sample size estimation

Minimally important differences in the primary outcome and the corresponding SD are defined as follows according to previous studies: 6-minute walk distance: difference of 26 m (SD 55m)[99]
Regarding secondary outcomes, our recent studies in healthy subjects and in patients with obstructive sleep apnea syndrome revealed the following changes at 2590 m compared to 490 m: a mean decrease in FVC of 6% (SD 7%); an increase in the BORG rating during the 6 min walk of 0.8 (1.6); a decrease in nocturnal oxygen saturation of 5% (2%).[100,101]
Power calculations indicate a required sample size of 45 COPD patients to detect the minimal differences listed above with a power of 80% at α 0.05. Assuming a drop-out rate of 10%, 50 participants will be recruited.

In patients with PH and ILD minimal important differences in the 6 min walk distance of 35-40 m have been reported.[102,103] In order to detect a difference of 40 m between values at 490 and 2702 m (SD 90m) with alpha 0.05 and power of 0.80 the required sample size is 42. Accounting for drop-outs the aim is to recruit 50 patients per group.

8.3 STATISTICAL ANALYSIS

Before analysis, data are anonymized by allocating a numerical code to each participant. Completeness, plausibility and distribution of data will be assessed by inspection of numerical results and graphical display. Normality of distribution will be tested by the Shapiro Wilks statistic. Data will be summarized by means (SD) and medians (quartiles) for normal and non-normal distributions. Effects of treatment and altitude will be evaluated according to the principles of cross-over trial analysis described by Senn S.[104] using ANOVA and multiple regression.

8.4 SIGNIFICANCE LEVEL

Statistical significance will be assumed if the null hypothesis is rejected with probability of $P<0.05$ applying a Bonferroni correction as appropriate.

8.5 HANDLING OF MISSING DATA

This will be performed as Little RJ et al[105]. All feasible measures will be taken to prevent drop-out of participants. In study phase 1 (effects of altitude) analysis will be performed as per protocol and missing data will be replaced by the group median for the corresponding outcome and altitude. In the study phase 2 (effects of oxygen) analysis will be performed according to the intention to treat principle and missing values will be replaced by the corresponding value during the alternative treatment conservatively assuming no treatment effect.

8.6 DEFINITION OF ANALYSIS PRINCIPLE

As mentioned above, in the study phase 1 (effects of altitude) the analyses will be according to protocol. In the study phase 2 (effects of oxygen) the analysis will be according to the intention to treat.

9 PRECAUTIONS AND DUTIES

9.1 SPECIFIC PRECAUTIONS

At all study locations, a medical doctor will be present and equipped with emergency material including bottled oxygen, drugs, defibrillator, ambu-bag, a ventilator for non-invasive mask ventilation. An evacuation plan will be prepared to bring patients to lower altitude if required (from Corvatsch Murtèl). Maximal bicycle exercise will be performed at 1721m only to avoid strenuous exercise at 2702m. Participants will be withdrawn from the study if they experience a deterioration of their underlying illness or new health threats (new diseases, accidents etc.).

Pregnant and nursing women will not be included. If a study participant will become pregnant, investigator have to be informed and participant is not allowed to continue the study. The course of the pregnancy and the birth should be reported to investigators. Patients should refrain from use of sedatives and alcohol and from strenuous exercise at 2702m.

10 DUTIES OF THE INVESTIGATOR

10.1 DECLARATION OF CONFORMITY

The study will be conducted in accordance with principles enunciated in the current Declaration of Helsinki, the guidelines of Good Clinical Practice (GCP) issued by ICH, and Swiss regulatory authority's requirements. Approval from the Ethics Committee will be obtained.
Consent to enter the study will be sought from each participant only after a full explanation has been given, an information leaflet offered and time allowed for consideration. Signed participant consent should be obtained. The right of the participant to refuse to participate without giving reasons must be respected.
After the participant has entered the study the clinician remains free to give alternative treatment to that specified in the protocol at any stage if he/she feels it is in the participant's best interest, but the reasons for doing so should be recorded. In these cases the participants remain within the study for the purposes of follow-up and data analysis. All participants are free to withdraw at any time from the protocol treatment without giving reasons and without prejudicing further treatment. The investigators will preserve the confidentiality of participants taking part in the study and is registered under the Data Protection Act.

10.2 REPORTING PROCEDURES

All adverse events will be reported. Depending on the nature of the event the reporting procedures below should be followed. Any questions concerning adverse event reporting will be directed to the Principal Investigator in the first instance. Non SAEs, whether expected or not, will be recorded. SAE's form will be completed and forwarded to the Principal Investigator within 24 hours. The Principal Investigator will assess whether the event is 'related', i.e. resulted from the administration of any of the research procedures; and 'unexpected', i.e. an event that is not listed in the protocol as an expected occurrence.
Reports of suspected related and unexpected SAEs (SUSARs) will be submitted within 15 days and within 7 days for fatal or life-threatening SUSARs, respectively, of the investigator becoming aware of the event, to the Ethics Committee using the CIOMS format.

The Ethics committee will be informed about changes in the protocol, interim and final reports will be delivered.

10.3 INSURANCE

Insurance is covered by "Betriebshaftpflichtversicherung des UniversitätsSpital Zürich".

So as not to forfeit their insurance cover, the subjects themselves must comply with the following conditions: Any deterioration in the subject's state of health that may have occurred as a result of the clinical trial must be reported immediately to the investigator, so he or she can notify the insurance company. The subject must take any appropriate measures that may help to determine the cause or the extent of damage, and to minimize the damage. In the event of a subject's death, the insurer must be notified immediately. The subject must not be involved in any other clinical trial during the course of this trial, nor within a period of 30 days prior to its beginning or 30 days after its completion.

Participants will be treated for adverse health effects during the study. An insurance of the University Hospital of Zurich covers any health-related damages. No cost will be charged to the patients who will participate to the study and the travel expense will be reimbursed.

11 ETHICAL CONSIDERATIONS

11.1 RISK BENEFIT ASSESSMENT

Millions of patients with COPD and other chronic lung diesases worldwide travel to moderate altitude for professional or recreational activities. In Switzerland and in other countries COPD patients are even referred for medical treatment in high altitude clinics such as the Hochgebirgsklinik Davos Wolfgang, the Zürcher Höhenklinik Davos Clavadel or the Luzerner and Berner Höhenkliniken in Montana. We expect that our inclusion and exclusion criteria will prevent that the study participants experience clinically relevant harm from the exposure to moderate altitude up to 2702 m. Precautions are taken to have all necessary measures to treat and evacuate patients if necessary.

The benefit of our study is to gain knowledge on health effects of altitude exposure in COPD, PH and ILD patients. This will help to counsel such patients planning altitude travel and to prevent altitude related illness in the future, which is of great value and outweighs the potential risks of the current study by far.

11.2 REASON FOR INCLUDING PATIENTS WITH COPD, PD AND ILD

The study purpose requires inclusions of patients with COPD, PH and ILD.

11.3 OTHER ETHICAL CONSIDERATIONS

None.

12 QUALITY CONTROL

The day-to-day management of the study will be coordinated by Prof. Dr. K. Bloch, Dr. med. T. Latshang.

12.1 DATA ACCESS, AUDITS, INSPECTIONS AND MONITORING

For each patient a Case Report Form (CRF) must be completed and signed by the principal investigator or authorized delegate from the study staff. This also applies to records for those patients who fail to complete the study. If a patient withdraws from the study, the reason must be noted on the CRF. If a patient is withdrawn from the study because of a treatment-limiting AE, thorough efforts should be made to clearly document the outcome. The trial master file will be documented and stored in electronic and paper form at the Klinik für Pneumologie. Zurich University Hospital in room C-RAE 34, and is therefore accessible to authorized medical and research staff only. The study may be subject to inspection and audit by regulatory bodies (public authorities, ethics commission), to ensure adherence to GCP, national law, and regulatory requirements. No other independent monitoring will be arranged.

12.2 CONFIDENTIALITY, ARCHIVING AND DESTRUCTION OF TRIAL DOCUMENTS AND PATIENT RECORDS

The investigator assures that subject' anonymity will be maintained and that their identities are protected from unauthorized parties. On CRFs or other documents, subjects should not be identified by their names, but by an identification code. The investigator keeps a subject enrolment log showing codes, names and addresses. The investigator maintains documents, e.g., subjects` written consent forms, in strict confidence. The informed consent form report a statement by which the patients allows the authorized personnel, the ethic committee and the regulatory authorities to have direct access to original medical records, which support the data on the CRFs. These personnel, bound by professional secrecy must maintain the confidentiality of al personal identity or personal medical information. Essential documents hall be archived safely and securely in such a way that ensures that they will be readily available upon the request from a regulatory authority. Data and all appropriate documentation will be stored for a minimum of 10 years after the completion of the study according to local regulations and in accordance with the maximum period of time permitted by the hospital.

12.3 DESCRIPTION OF DATA IN THE CRFS

Questionnaires, clinical examination, and 6-minute walking test will be reported in the case report forms.

13 PUBLICATION POLICY

The study's results will be presented as scientific papers in medical or physiologic publications.

14 REFERENCE LIST

1. GOLD Executive Committee. Global strategy for diagnosis, management, and prevention of COPD. 2011.
2. Bridevaux PO et al. Prevalence of airflow obstruction in smokers and never-smokers in Switzerland. Eur Respir J 2010;36:1259-1269.

3. Niewoehner DE et al. Effect of systemic glucocorticoids on exacerbations of chronic obstructive pulmonary disease. Department of Veterans Affairs Cooperative Study Group. N Engl J Med 1999;340:1941-1947.

4. Bloch KE et al. Gain and subsequent loss of lung function after lung volume reduction surgery in cases of severe emphysema with different morphologic patterns. J Thorac Cardiovasc Surg 2002;123:845-854.

5. Tutic M et al. Lung-volume reduction surgery as an alternative or bridging procedure to lung transplantation. Ann Thorac Surg 2006;82:208-213.

6. Galie N et al. Guidelines for the diagnosis and treatment of pulmonary hypertension: the Task Force for the Diagnosis and Treatment of Pulmonary Hypertension of the European

Society of Cardiology (ESC) and the European Respiratory Society (ERS), endorsed by the International Society of Heart and Lung Transplantation (ISHLT). Eur Heart J 2009;30:2493-2537.

7. Montani D et al. Pulmonary arterial hypertension. Orphanet J Rare Dis 2013;8:97.

8. Cenedese E et al. Measurement of quality of life in pulmonary hypertension and its significance. Eur Respir J 2006;28:808-815.

9. Montani D et al. Targeted therapies in pulmonary arterial hypertension. Pharmacol Ther 2013.

10. Travis WD et al. An official American Thoracic Society/European Respiratory Society statement: Update of the international multidisciplinary classification of the idiopathic interstitial pneumonias. Am J Respir Crit Care Med 2013;188:733-748.

11. Antoniou KM et al. Pharmacological treatment of idiopathic pulmonary fibrosis: from the past to the future. Eur Respir Rev 2013;22:281-291.

12. Karrer W et al. [Nasal CPAP therapy in obstructive sleep apnea syndrome: patient compliance]. Schweiz Med Wochenschr 2000;130:1291-1297.

13. Speelberg B et al. [Lung function of adult patients with bronchial asthma or chronic obstructive lung disease prior to and following a 3-month-stay in the Dutch Asthma Center in Davos]. Ned Tijdschr Geneeskd 1992;136:469-473.

14. Bijl D et al. Pulmonary rehabilitation at moderate altitude: a 1-year follow-up. Neth J Med 1994;45:154-161.

15. Dempsey JA. Crossing the apnoeic threshold: causes and consequences. Exp Physiol 2005;90:13-24.

16. Bloch KE et al. Nocturnal periodic breathing during acclimatization at very high altitude at Mount Muztagh Ata (7,546 m). Am J Respir Crit Care Med 2010;182:562-568.

17. Latshang TD. Are sleep. nocturnal breathing and daytime performance impaired at moderate altitude? Respiration 2011;82:76.

18. Nussbaumer-Ochsner Y et al. Effect of short-term acclimatization to high altitude on sleep and nocturnal breathing. Sleep 2012;35:419-423.

19. Deboeck G et al. Respiratory muscle strength may explain hypoxia-induced decrease in vital capacity. Med Sci Sports Exerc 2005;37:754-758.

20. Senn O et al. Do changes in lung function predict high-altitude pulmonary edema at an early stage? Med Sci Sports Exerc 2006;38:1565-1570.

21. Bartsch P et al. Physiological aspects of high-altitude pulmonary edema. J Appl Physiol 2005;98:1101-1110.

22. Nussbaumer-Ochsner Y et al. Lessons from high-altitude physiology. Breathe 2007;4:123-132.

23. Maggiorini M et al. Prevalence of acute mountain sickness in the Swiss Alps. BMJ 1990;301:853-855.

24. Bloch KE et al. Effect of ascent protocol on acute mountain sickness and success at Muztagh Ata, 7546 m. High Alt Med Biol 2009;10:25-32.

25. Erba P et al. Acute mountain sickness is related to nocturnal hypoxemia but not to hypoventilation. Eur Respir J 2004;24:303-308.

26. Maggiorini M et al. Both tadalafil and dexamethasone may reduce the incidence of high-altitude pulmonary edema: a randomized trial. Ann Intern Med 2006;145:497-506.

27. Muhm JM et al. Effect of aircraft-cabin altitude on passenger discomfort. N Engl J Med 2007;357:18-27.

28. Dillard TA et al. Lung function during moderate hypobaric hypoxia in normal subjects and patients with chronic obstructive pulmonary disease. Aviat Space Environ Med 1998;69:979-985.

29. Christensen CC et al. Development of severe hypoxaemia in chronic obstructive pulmonary disease patients at 2,438 m (8,000 ft) altitude. Eur Respir J 2000;15:635-639.

30. Akero A et al. Pulse oximetry in the preflight evaluation of patients with chronic obstructive pulmonary disease. Aviat Space Environ Med 2008;79:518-524.

31. Seccombe LM et al. Effect of simulated commercial flight on oxygenation in patients with interstitial lung disease and chronic obstructive pulmonary disease. Thorax 2004;59:966-970.

32. Chetta A et al. Walking capacity and fitness to fly in patients with chronic respiratory disease. Aviat Space Environ Med 2007;78:789-792.

33. Dillard TA et al. Hypoxemia during altitude exposure. A meta-analysis of chronic obstructive pulmonary disease. Chest 1993;103:422-425.

34. Muhm JM. Predicted arterial oxygenation at commercial aircraft cabin altitudes. Aviat Space Environ Med 2004;75:905-912.

35. Bradi AC et al. Predicting the need for supplemental oxygen during airline flight in patients with chronic pulmonary disease: a comparison of predictive equations and altitude simulation. Can Respir J 2009;16:119-124.

36. Ahmedzai S et al. Managing passengers with stable respiratory disease planning air travel: British Thoracic Society recommendations. Thorax 2011;66 Suppl 1:i1-30.

37. Shrikrishna D et al. Managing passengers with stable respiratory disease planning air travel: British Thoracic Society recommendations. Thorax 2011;66:831-833.

38. Edvardsen A et al. High prevalence of respiratory symptoms during air travel in patients with COPD. Respir Med 2011;105:50-56.

39. Coker RK et al. Is air travel safe for those with lung disease? Eur Respir J 2008;32:1423-1424.

40. Akero A et al. Hypoxaemia in chronic obstructive pulmonary disease patients during a commercial flight. Eur Respir J 2005;25:725-730.

41. Kelly PT et al. Air travel hypoxemia vs. the hypoxia inhalation test in passengers with COPD. Chest 2008;133:920-926.

42. FINKELSTEIN S et al. PULMONARY MECHANICS AT ALTITUDE IN NORMAL AND OBSTRUCTIVE LUNG DISEASE PATIENTS. Aerosp Med 1965;36:880-884.

43. Heindl S et al. Marked sympathetic activation in patients with chronic respiratory failure. Am J Respir Crit Care Med 2001;164:597-601.

44. Karrer W et al. [Respiration of patients with chronic lung disease at 500 and 1500 meter above sea level]. Schweiz Med Wochenschr 1990;120:1584-1589.

45. Kelly PT et al. Resting and exercise response to altitude in patients with chronic obstructive pulmonary disease. Aviat Space Environ Med 2009;80:102-107.

46. Graham WG et al. Short-term adaptation to moderate altitude. Patients with chronic obstructive pulmonary disease. JAMA 1978;240:1491-1494.

47. Luks AM et al. Travel to high altitude with pre-existing lung disease. Eur Respir J 2007;29:770-792.

48. Akero A et al. COPD and air travel: oxygen equipment and preflight titration of supplemental oxygen. Chest 2011;140:84-90.

49. Basnyat B et al. Efficacy of low-dose acetazolamide (125 mg BID) for the prophylaxis of acute mountain sickness: a prospective, double-blind, randomized, placebo-controlled trial. High Alt Med Biol 2003;4:45-52.

50. Latshang TD et al. Does autoCPAP therapy control breathing disturbances in patients with obstructive sleep apnea syndrome at altitude? JAMA 2012.

51. Nussbaumer-Ochsner Y et al. Patients with obstructive sleep apnea syndrome benefit from acetazolamide during an altitude sojourn: a randomized, placebo-controlled, double-blind trial. Chest 2012;141:131-138.

52. Fischler M et al. Dexamethasone but not tadalafil improves exercise capacity in adults prone to high-altitude pulmonary edema. Am J Respir Crit Care Med 2009;180:346-352.

53. Murata T et al. Dexamethasone blocks hypoxia-induced endothelial dysfunction in organ-cultured pulmonary arteries. Am J Respir Crit Care Med 2004;170:647-655.

54. Scherrer U et al. Suppression of insulin-induced sympathetic activation and vasodilation by dexamethasone in humans. Circulation 1993;88:388-394.

55. Chaouat A et al. Pulmonary hypertension in COPD. Eur Respir J 2008;32:1371-1385.

56. Richalet JP et al. Sildenafil inhibits altitude-induced hypoxemia and pulmonary hypertension. Am J Respir Crit Care Med 2005;171:275-281.

57. Ghofrani HA et al. Sildenafil increased exercise capacity during hypoxia at low altitudes and at Mount Everest base camp: a randomized, double-blind, placebo-controlled crossover trial. Ann Intern Med 2004;141:169-177.

58. Blanco I et al. Hemodynamic and gas exchange effects of sildenafil in patients with chronic obstructive pulmonary disease and pulmonary hypertension. Am J Respir Crit Care Med 2010;181:270-278.

59. Luks AM et al. Travel to high altitude with pre-existing lung disease. Eur Respir J 2007;29:770-792.

60. Naeije R et al. High-altitude pulmonary edema with primary pulmonary hypertension. Chest 1996;110:286-289.

61. Burns RM et al. Hypoxaemia in patients with pulmonary arterial hypertension during simulated air travel. Respir Med 2013;107:298-304.

62. Thamm M et al. Air travel can be safe and well tolerated in patients with clinically stable pulmonary hypertension. Pulm Circ 2011;1:239-243.

63. Roubinian N et al. Effects of commercial air travel on patients with pulmonary hypertension air travel and pulmonary hypertension. Chest 2012;142:885-892.

64. Ahmedzai S et al. Managing passengers with stable respiratory disease planning air travel: British Thoracic Society recommendations. Thorax 2011;66 Suppl 1:i1-30.

65. Christensen CC et al. Effect of hypobaric hypoxia on blood gases in patients with restrictive lung disease. Eur Respir J 2002;20:300-305.

66. Coker RK et al. Is air travel safe for those with lung disease? Eur Respir J 2007;30:1057-1063.

67. Nussbaumer-Ochsner Y et al. Exacerbation of sleep apnoea by frequent central events in patients with the obstructive sleep apnoea syndrome at altitude: a randomised trial. Thorax 2010;65:429-435.

68. ATS statement: guidelines for the six-minute walk test. Am J Respir Crit Care Med 2002;166:111-117.

69. Fitzgerald RS et al. Effect of hypoxia on carotid chemoreceptor response to carbon dioxide in cats. Respir Physiol 1971;12:218-229.

70. Jones PW et al. Development and first validation of the COPD Assessment Test. Eur Respir J 2009;34:648-654.

71. Borg E et al. A comparison between three rating scales for perceived exertion and two different work tests. Scand J Med Sci Sports 2006;16:57-69.

72. Mahler DA et al. Evaluation of clinical methods for rating dyspnea. Chest 1988;93:580-586.

73. Sampson JB et al. Procedures for the measurement of acute mountain sickness. Aviat Space Environ Med 1983;54:1063-1073.

74. Bloch KE et al. German version of the Epworth Sleepiness Scale. Respiration 1999;66:440-447.

75. Hoddes E et al. Quantification of sleepiness: a new approach. Psychophysiology 1973;10:431-436.

76. Miller MR et al. Standardisation of spirometry. Eur Respir J 2005;26:319-338.

77. MacIntyre N et al. Standardisation of the single-breath determination of carbon monoxide uptake in the lung. Eur Respir J 2005;26:720-735.

78. Basner M et al. Maximizing sensitivity of the psychomotor vigilance test (PVT) to sleep loss. Sleep 2011;34:581-591.

79. Tombaugh TN. Trail Making Test A and B: normative data stratified by age and education. Arch Clin Neuropsychol 2004;19:203-214.

80. Weber KP et al. Head impulse test in unilateral vestibular loss: vestibulo-ocular reflex and catch-up saccades. Neurology 2008;70:454-463.

81. Weber KP et al. Horizontal head impulse test detects gentamicin vestibulotoxicity. Neurology 2009;72:1417-1424.

82. MacDougall HG et al. The video head impulse test: diagnostic accuracy in peripheral vestibulopathy. Neurology 2009;73:1134-1141.

83. Weber KP et al. Impulsive testing of semicircular-canal function using video-oculography. Ann N Y Acad Sci 2009;1164:486-491.

84. Cymerman A et al. Saccadic velocity and pupillary reflexes during acclimatization to altitude (4300 m). Aviat Space Environ Med 2005;76:627-634.

85. Munoz DP et al. Look away: the anti-saccade task and the voluntary control of eye movement. Nat Rev Neurosci 2004;5:218-228.

86. Abel LA et al. End-point nystagmus. Invest Ophthalmol Vis Sci 1978;17:539-544.

87. Shallo-Hoffmann J et al. A reexamination of end-point and rebound nystagmus in normals. Invest Ophthalmol Vis Sci 1990;31:388-392.

88. Lang RM et al. Recommendations for chamber quantification: a report from the American Society of Echocardiography's Guidelines and Standards Committee and the Chamber Quantification Writing Group, developed in conjunction with the European Association of Echocardiography, a branch of the European Society of Cardiology. J Am Soc Echocardiogr 2005;18:1440-1463.

89. Abbas AE et al. A simple method for noninvasive estimation of pulmonary vascular resistance. J Am Coll Cardiol 2003;41:1021-1027.

90. Imholz BP et al. Feasibility of ambulatory, continuous 24-hour finger arterial pressure recording. Hypertension 1993;21:65-73.

91. Katayama K et al. Muscle deoxygenation during sustained and intermittent isometric exercise in hypoxia. Med Sci Sports Exerc 2010;42:1269-1278.

92. Ainslie PN et al. Differential responses to CO2 and sympathetic stimulation in the cerebral and femoral circulations in humans. J Physiol 2005;566:613-624.

93. Poulin MJ et al. Fast and slow components of cerebral blood flow response to step decreases in end-tidal PCO2 in humans. J Appl Physiol 1998;85:388-397.

94. Ide K et al. Relationship between middle cerebral artery blood velocity and end-tidal PCO2 in the hypocapnic-hypercapnic range in humans. J Appl Physiol 2003;95:129-137.

95. Claassen JA et al. Transcranial Doppler estimation of cerebral blood flow and cerebrovascular conductance during modified rebreathing. J Appl Physiol 2007;102:870-877.

96. Kohler M et al. Children at high altitude have less nocturnal periodic breathing than adults. Eur Respir J 2008;32:189-197.

97. Thurnheer R et al. Accuracy of nasal cannula pressure recordings for assessment of ventilation during sleep. Am J Respir Crit Care Med 2001;164:1914-1919.

98. Maarsingh EJ et al. Respiratory muscle activity measured with a noninvasive EMG technique: technical aspects and reproducibility. J Appl Physiol 2000;88:1955-1961.

99. Puhan MA et al. Interpretation of treatment changes in 6-minute walk distance in patients with COPD. Eur Respir J 2008;32:637-643.

100. Latshang TD et al. Are Nocturnal Breathing, Sleep, and Cognitive Performance Impaired at Moderate Altitude (1,630-2,590 m)? Sleep 2013;36:1969-1976.

101. Latshang TD et al. Effect of acetazolamide and autoCPAP therapy on breathing disturbances among patients with obstructive sleep apnea syndrome who travel to altitude: a randomized controlled trial. JAMA 2012;308:2390-2398.

102. du Bois RM et al. Six-minute-walk test in idiopathic pulmonary fibrosis: test validation and minimal clinically important difference. Am J Respir Crit Care Med 2011;183:1231-1237.

103. Mathai SC et al. The minimal important difference in the 6-minute walk test for patients with pulmonary arterial hypertension. Am J Respir Crit Care Med 2012;186:428-433.

104. Senn S. Cross-over trials in clinical research. 2002;2:1-344.

105. Little RJ et al. The prevention and treatment of missing data in clinical trials. N Engl J Med 2012;367:1355-1360.

15 APPENDICES

- Patient Information Sheet/ Informed Consent Form
- Case Report Forms (CRF)
- Public study advertisement
- Study protocol summary in German
- Electronic Version of the protocol and further documentation on CD-ROM
- Report on study phase 1 in COPD patients June-October 2013
