# Supplementary material for: Effect of Nocturnal Oxygen Therapy on Nocturnal Hypoxemia and Sleep Apnea Among Patients With Chronic Obstructive Pulmonary Disease Traveling to 2048 Meters: A Randomized Clinical Trial
Source: JAMA Netw Open. 2020 Jun 22;3(6):e207940. doi: 10.1001/jamanetworkopen.2020.7940 (PMC7309443; doi:10.1001/jamanetworkopen.2020.7940)
Supplement: Supplement 2. — eAppendix. Supplementary Methods eTable 1. Mixed Linear Regression for the Coprimary Outcome of Mean Nocturnal Oxygen Saturation Including Location, Intervention, and Randomized Exposure Sequence eTable 2. Mixed Linear Regression for the Coprimary Outcome of Apnea-Hypopnea Index Including Location, Intervention, and Randomized Exposure Sequence eTable 3. Mixed Linear Regression Models of the Apnea-Hypopnea Index at 2048 m Under Placebo Intervention eTable 4. Mixed Linear Regression Models of Mean Nocturnal Oxygen Saturation at 2048 m Under Placebo Intervention eTable 5. Logistic Regression Analyses of Potential Variables Associated With Altitude-Related Adverse Health Effects (ARAHE at 2048 m) eReferences. [file jamanetwopen-3-e207940-s002.pdf]

Supplementary Online Content

Tan L, Latshang TD, Aeschbacher SS, et al. Effect of nocturnal oxygen therapy on nocturnal hypoxemia and sleep apnea among patients with chronic obstructive pulmonary disease traveling to 2048 meters: a randomized clinical trial. *JAMA Netw Open*. 2020;3(6):e207940. doi:10.1001/jamanetworkopen.2020.7940

eAppendix. Supplementary Methods
eTable 1. Mixed Linear Regression for the Coprimary Outcome of Mean Nocturnal Oxygen Saturation Including Location, Intervention, and Randomized Exposure Sequence
eTable 2. Mixed Linear Regression for the Coprimary Outcome of Apnea-Hypopnea Index Including Location, Intervention, and Randomized Exposure Sequence
eTable 3. Mixed Linear Regression Models of the Apnea-Hypopnea Index at 2048 m Under Placebo Intervention
eTable 4. Mixed Linear Regression Models of Mean Nocturnal Oxygen Saturation at 2048 m Under Placebo Intervention
eTable 5. Logistic Regression Analyses of Potential Variables Associated With Altitude-Related Adverse Health Effects (ARAHE at 2048 m)
eReferences.

This supplementary material has been provided by the authors to give readers additional information about their work.

© 2020 Tan L et al. *JAMA Network Open.*

eAppendix. Supplementary Methods

Patients – Exclusion criteria

Patients with unstable condition, COPD exacerbation within the last 4 months before the study, very severe COPD (GOLD 4, FEV_1 <30% predicted), hypoxemia at low altitude (SpO_2 <92% or using oxygen therapy), uncontrolled cardiovascular disease, history of obstructive sleep syndrome, use of drugs that affect respiratory center drive, previous intolerance of moderate altitude (<2600 m) or exposure to altitudes >1500 m for >2 days within the last 4 weeks before the study were excluded.

Assessments

In the first night of each visit, polysomnography (Alice 5, Philips Respironics, Zofingen, Switzerland) was performed according to international standards.[1,2] Electroencephalogram (C3A1, C4A2), bilateral electro-oculogram, submental electromyogram, pulse oximetry, nasal pressure swings, calibrated respiratory inductance plethysmography, diaphragmatic surface EMG, transcutaneous capnography (PtcCO2) and cerebral tissue oxygen saturation (CTO) by near-infrared spectroscopy (NIRS) were recorded.[3]

Sleep stages and arousals were scored according to Rechtschaffen and Kales standard[4] and American Academy of Sleep Medicine.[2] Apneas/hypopneas were defined as a reduction of nasal pressure swings or the inductive plethysmographic sum signal to <50 % of baseline for >10 seconds.[5] Transient reductions in breathing amplitude to <50 % of baseline for 5-10 seconds were also scored as apneas/hypopneas if they occurred as part of a periodic breathing pattern with hyperventilation alternating with central apneas/hypopneas for at least three consecutive cycles. Obstructive apneas/hypopneas were identified by rib cage/abdominal asynchrony or paradoxical motion, deformation of nasal pressure curves suggesting inspiratory flow limitation (flattening), or increasing diaphragmatic EMG activity. Central apneas/hypopneas were defined as absent rib cage and abdominal asynchrony, no signs of inspiratory flow limitations (no flattening of nasal pressure contour) and reduced or absent diaphragmatic EMG activity.[5] Mixed apneas/hypopneas were classified as obstructive events.

Arterial blood gas analysis and measurement of hemoglobin concentration were performed on a radial artery blood sample drawn in the morning during ambient air breathing (RapidPoint 405, Siemens Healthcare Diagnostics AG, Zurich, Switzerland).

The 6-min walk distance (6MWD) was measured in the morning after the first night of each visit. Dyspnea sensation was rated at the end of test on a Borg CR10 scale.[6] Lung function tests were performed according to ATS/ERS guidelines.[7] Single breath diffusing capacity for carbon monoxide (DLCO) was adjusted for hemoglobin and predicted values computed without and with adjustment for altitude. The equation used for the adjustment for altitude was DLCO, predicted for altitude = DLCO, predicted/(1.0+0.0031 (PI, O2-150)).[8]

© 2020 Tan L et al. *JAMA Network Open.*

eTable 1. Mixed Linear Regression for the Coprimary Outcome of Mean Nocturnal Oxygen Saturation Including Location, Intervention, and Randomized Exposure Sequence

Dependent variable: Mean nocturnal oxygen saturation (SpO_2)

**Covariates**	**Coef.**	**Std. Err**	**P-value**	**95% CI**
SpO_2 at 490m (reference)	91.4	1.0	<.001	89.4 to 93.2
Δ night 1 at 2048m room air vs. 490m	-6.1	0.6	<.001	-7.2 to -5.0
Δ night 2 2048m room air vs. 490m	-5.1	0.6	<.001	-6.2 to -4.0
Δ night 1 at 2048m NOT vs. 490m	4.1	0.6	<.001	3.0 to 5.3
Δ night 2 2048m NOT vs. 490m	3.4	0.6	<.001	2.3 to 4.6
Randomized exposure sequence (1-4)	0.4	0.3	.25	-0.3 to 1.0

SpO_2 = nocturnal arterial oxygen saturation assessed by finger oximetry; NOT = nocturnal nasal oxygen therapy.

© 2020 Tan L et al. *JAMA Network Open.*

eTable 2. Mixed Linear Regression for the Coprimary Outcome of Apnea-Hypopnea Index Including Location, Intervention, and Randomized Exposure Sequence

Dependent variable: Apnea-hypopnea index during time in bed (AHI)				
**Covariates**	**Coef.**	**Std. Err**	**P-value**	**95% CI**
AHI at 490m (reference)	17.6	8.0	.03	1.9 to 33.2
Δ night 1 at 2048m room air vs. 490m	16.3	3.8	<.001	8.8 to 23.8
Δ night 2 2048m room air vs. 490m	7.6	3.8	.05	0.1 to 15.0
Δ night 1 at 2048m NOT vs. 490m	-5.5	3.8	.15	-13.0 to 2.0
Δ night 2 2048m NOT vs. 490m	-9.4	3.8	.01	-16.9 to -1.9
Randomized exposure sequence (1-4)	0.9	2.8	.75	-4.5 to 6.3

AHI = apnea-hypopnea index; NOT = nocturnal nasal oxygen therapy.

© 2020 Tan L et al. *JAMA Network Open.*

eTable 3. Mixed Linear Regression Models of the Apnea-Hypopnea Index at 2048 m Under Placebo Intervention

Dependent variable: Apnea-hypopnea index during time in bed (AHI)				
**Variables**	**Coef.**	**Std. Err**	**P-value**	**95% CI**
AHI at 490 m, 1/hour TIB	0.65	0.33	.05	0.00 to 1.30
Age, years	0.29	0.92	.75	-1.51 to 2.09
Female vs. male	-17.45	9.06	.05	-35.21 to 0.31
Body mass index at 490 m, kg/m^2	0.63	1.10	.57	-1.53 to 2.79
FEV_1 at 490 m, % predicted	0.24	0.36	.51	-0.46 to 0.94
2nd vs, 1st night at 2048 m	-8.75	2.65	.001	-13.93 to -3.56
Randomized exposure sequence (1-4)	0.97	3.83	.80	-6.55 to 8.48
Intercept	9.17	73.27	.90	-134.44 to 152.77

Predictors were assessed at 490 m, otherwise stated. AHI = apnea-hypopnea index; FEV_1 = forced expiratory volume in the first second of expiration.

© 2020 Tan L et al. *JAMA Network Open.*

eTable 4. Mixed Linear Regression Models of Mean Nocturnal Oxygen Saturation at 2048 m Under Placebo Intervention

Dependent variable: Nocturnal arterial oxygen saturation (SpO_2)				
**Variables**	**Coef.**	**Std. Err**	**P-value**	**95% CI**
Nocturnal SpO_2 at 490 m, %	1.01	0.20	<.001	0.61 to 1.40
Age, years	0.12	0.08	.14	-0.04 to 0.28
Female vs. male	-1.40	0.74	.06	-2.85 to 0.05
Body mass index at 490 m, kg/m^2	0.05	0.09	.60	-0.13 to 0.23
FEV_1 at 490 m, % predicted	0.01	0.03	.65	-0.05 to 0.07
2nd vs, 1st night at 2048 m	1.00	0.50	.04	0.03 to 1.97
Randomized exposure sequence (1-4)	0.00	0.33	.99	-0.64 to 0.65
Intercept	-15.59	22.04	.48	-58.79 to 27.60

Predictors were assessed at 490 m, otherwise stated. SpO_2 = nocturnal arterial oxygen saturation assessed by finger oximetry; FEV_1 = forced expiratory volume in the first second of expiration.

© 2020 Tan L et al. *JAMA Network Open.*

eTable 5. Logistic Regression Analyses of Potential Variables Associated With Altitude-Related Adverse Health Effects (ARAHE at 2048 m)

Dependent variable: Altitude-related adverse health effects (0 = no, 1 = yes)

Predictors assessed at 490 m	Odds ratio	Std. Err	P-value	95% CI
Age, years	0.96	0.07	.51	0.83 to 1.09
Female vs Male	0.62	0.49	.54	0.13 to 2.90
Body mass index, kg/m^2	1.13	0.11	.21	0.93 to 1.37
FEV_1, %predicted	0.99	0.03	.67	0.93 to 1.05
FEV_1 / FVC ratio	0.98	0.04	.56	0.90 to 1.06
DLCO, %predicted	0.97	0.03	.23	0.92 to 1.02
Nocturnal SpO_2, %	0.59	0.16	.05	0.34 to 1.01
Apnoea/hypopnoea index, 1/h	1.04	0.04	.22	0.97 to 1.12
Daytime SpO_2, %	0.36	0.19	.05	0.13 to 1.02
SaO_2, %	0.89	0.28	.71	0.48 to 1.65
PaO_2, kPa	0.55	0.39	.39	0.14 to 2.18
$PaCO_2$, kPa	0.48	0.50	.48	0.06 to 3.62
6-minute walk distance, m	0.99	0.01	.08	0.98 to 1.00
Current vs. former smoker	2.88	2.42	.21	0.56 to 14.94
Smoking pack-years	1.00	0.01	.86	0.97 to 1.02

Predictors were assessed at 490 m. All patients had a smoking history. FEV_1 = forced expiratory volume in the first second of expiration; FVC = forced vital capacity; DLCO = diffusing capacity for carbon monoxide; SpO_2 = nocturnal arterial oxygen saturation assessed by finger oximetry; SaO_2, arterial oxygen saturation; PaO_2 = PaO_2, partial pressure of arterial O_2; $PaCO_2$ = partial pressure of arterial CO_2

© 2020 Tan L et al. *JAMA Network Open.*

eReferences

1. Latshang TD, Nussbaumer-Ochsner Y, Henn RM, et al. Effect of acetazolamide and autoCPAP therapy on breathing disturbances among patients with obstructive sleep apnea syndrome who travel to altitude: a randomized controlled trial. *JAMA* 2012; **308**(22): 2390-8.
2. EEG arousals: scoring rules and examples: a preliminary report from the Sleep Disorders Atlas Task Force of the American Sleep Disorders Association. *Sleep* 1992; **15**(2): 173-84.
3. Schwarz EI, Furian M, Schlatzer C, Stradling JR, Kohler M, Bloch KE. Nocturnal cerebral hypoxia in obstructive sleep apnoea: a randomised controlled trial. *Eur Respir J* 2018; **51**(5).
4. Hori T, Sugita Y, Koga E, et al. Proposed supplements and amendments to 'A Manual of Standardized Terminology, Techniques and Scoring System for Sleep Stages of Human Subjects', the Rechtschaffen & Kales (1968) standard. *Psychiatry Clin Neurosci* 2001; **55**(3): 305-10.
5. Nussbaumer-Ochsner Y, Schuepfer N, Ulrich S, Bloch KE. Exacerbation of sleep apnoea by frequent central events in patients with the obstructive sleep apnoea syndrome at altitude: a randomised trial. *Thorax* 2010; **65**(5): 429-35.
6. Borg G. Borg's perceived exertion and pain scales. Champaign, IL: Human kinetics; 1998.
7. Miller MR, Hankinson J, Brusasco V, et al. Standardisation of spirometry. *Eur Respir J* 2005; **26**(2): 319-38.
8. MacIntyre NR, Nadel JA. Regional diffusing capacity in normal lungs during a slow exhalation. *J Appl Physiol Respir Environ Exerc Physiol* 1982; **52**(6): 1487-92.

© 2020 Tan L et al. *JAMA Network Open.*
