# Supplementary material for: Effect of Nocturnal Oxygen Therapy on Nocturnal Hypoxemia and Sleep Apnea Among Patients With Chronic Obstructive Pulmonary Disease Traveling to 2048 Meters: A Randomized Clinical Trial
Source: JAMA Netw Open. 2020 Jun 22;3(6):e207940. doi: 10.1001/jamanetworkopen.2020.7940 (PMC7309443; doi:10.1001/jamanetworkopen.2020.7940)
Supplement: Supplement 3. — Data Sharing Statement [file jamanetwopen-3-e207940-s003.pdf]

Data Sharing Statement

Tan. Effect of Nocturnal Oxygen Therapy on Nocturnal Hypoxemia and Sleep Apnea Among Patients With Chronic Obstructive Pulmonary Disease Traveling to 2048 Meters. *JAMA Netw Open*. Published June 22, 2020. 10.1001/jamanetworkopen.2020.7940

Data
Data available: Yes
Data types: Deidentified participant data
How to access data: michael.furian@usz.ch
When available: With publication

Supporting Documents
Document types: None

Additional Information
Who can access the data: Anonymized data underlying this study can be requested by qualified researchers providing an approved proposal.
Types of analyses: for any purpose
Mechanisms of data availability: Data will be made available only by approval of all investigators of this publication
Any additional restrictions: -
